# The NBGENS project protocol: Pilot study for genetic newborn screening in Galicia

**DOI:** 10.1371/journal.pone.0359270

**Published:** 2026-10-08

**Authors:** Lluis Lis-López, Catalina López-López, Laura López-Valverde, Salomé Quintáns-Lago, Bruno K. Rodiño-Janeiro, J. Víctor Álvarez, Francisco Barros, Sofía Gouveia, Mariana Serres-Gómez, Judit García-Villoria, María E. Vázquez-Mosquera, María Luz Couce

**Affiliations:** 1 Health Research Institute of Santiago de Compostela (IDIS), Santiago de Compostela, Spain; 2 Department of Forensic Sciences, Pathology, Gynecology and Obstetrics, Pediatrics, Neonatology Service, Hospital Clínico Universitario de Santiago de Compostela, CIBERER, MetabERN, RICORS-SAMID, Santiago de Compostela, Spain; 3 Xenomics Medicine Group, CIBERER, Galician Public Foundation of Xenomics Medicine - SERGAS, Santiago de Compostela, Spain; 4 Bijvoet Centre for Biomolecular Research, Utrecht University, Tilburg, the Netherlands; 5 Division of Inborn Errors of Metabolism-IBC, Department of Biochemistry and Molecular Genetics, CDB, Hospital Clinic de Barcelona, IDIBAPS, Barcelona, Spain; 6 Center for biomedical network research on rare diseases (CIBERER), ISCIII, Madrid, Spain; Government College University Faisalabad, PAKISTAN

## Abstract

**Background:**

Conventional newborn screening (NBS) relies on mainly biochemical assays and remains limited by its dependence on detectable biomarkers within a narrow postnatal window, leaving many treatable genetic disorders unidentified. Advances in next-generation sequencing have enabled projects of genomic newborn screening, which may expand the spectrum of detectable conditions. However, challenges persist regarding variant interpretation, reporting criteria, and integration into public healthcare systems.

**Methods:**

NBGenS is a prospective, single-center pilot study in Spain that will evaluate the feasibility of integrating whole exome sequencing (WES) into routine NBS. The study will recruit 550 newborns at the University Clinical Hospital of Santiago de Compostela. DNA will be extracted from residual dried blood spots (DBS) obtained through NBS. WES and clinical interpretation will be restricted to a virtual panel of genes associated with severe, highly-penetrant, pediatric-onset diseases for which early intervention is available. Variant interpretation will follow ACMG/AMP and ClinGen recommendations, and only pathogenic or likely pathogenic variants will be considered reportable. Untargeted metabolomics using DBS will be applied in selected cases, particularly for prioritised variants of uncertain significance (VUS), to provide complementary biochemical evidence that may contribute to variant classification. Clinical follow-up, psychosocial assessment, pharmacogenetic analysis, and health economic evaluation will also be incorporated into the study design.

**Outcomes:**

The project will establish and evaluate a comprehensive clinical workflow encompassing recruitment, sequencing, interpretation, reporting, and follow-up. Key outcomes will include diagnostic yield, parental acceptance, psychological impact, cost-effectiveness, clinical utility, and the added value of metabolomic integration for resolving VUS.

**Conclusion:**

NBGenS represents one of the first prospective public healthcare pilot studies integrating genomic sequencing and untargeted metabolomics within an established NBS program. The study will generate empirical evidence on the clinical, ethical, psychosocial, and economic implications of neonatal genomic screening and may inform future policy decisions regarding its implementation within national health systems.

## Introduction

Newborn screening (NBS) is one of the most effective and widely established public health interventions. Traditional NBS (tNBS) programs rely primarily on biochemical assays to detect altered biomarkers strongly associated with specific conditions, mainly metabolic and endocrine disorders [1–3]. Since their implementation worldwide, NBS programs have shown marked heterogeneity, reflecting differences in healthcare organization and economic resources across countries and regions [4,5]. Over time, these programs have expanded to include additional conditions that meet strict inclusion criteria, alongside the identification of increasingly sensitive and specific biochemical markers [6,7]. However, tNBS remains intrinsically limited in its ability to detect around 50 diseases due to its dependence on biomarkers detectable in dried blood spot (DBS) samples within a narrow postnatal window, leaving many rare genetic disorders with available treatments or preventive interventions undetected [8,9].

Advances in next-generation sequencing (NGS) and the expanding understanding of the human genome have enabled deeper insights into the molecular basis of genetic diseases, fostering multiple studies on genomic sequencing in both healthy and affected newborns. Early initiatives began in 2013 through projects funded in the United States by the Newborn Sequencing in Genomic Medicine and Public Health (NSIGHT) consortium, supported by the Eunice Kennedy Shriver National Institute of Child Health and Human Development (NICHD) and the National Human Genome Research Institute (NHGRI) [10]. These projects aimed to assess how genomic sequencing could improve healthcare outcomes for newborns with genetic conditions.

Different whole exome sequencing (WES)-based approaches were explored, including NBSeq, which targeted a virtual panel of 78 genes associated with inborn errors of metabolism (IEMs) included in tNBS programs [11]; NEXUS, which analyzed a virtual panel of 466 genes in healthy newborns and in those with IEMs or hearing loss detected through standard screening [12]; BabySeq, which evaluated a virtual panel of 954 genes in cohorts of healthy and affected newborns [13,14]. BabyDetect, which analyzed a custom target panel covering 405 genes [15]; and the study published by Shubina et al.[16],, which considered a virtual panel of 2,350 genes. The wide variation in gene content across these programs reflects the limited international consensus, driven by differences in actionability thresholds, disease severity, age of onset, and availability of effective interventions [17,18].

NBSeq demonstrated that WES analysis alone lacks sufficient sensitivity and specificity to serve as a primary screening tool for most IEMs but is valuable as a secondary test by identifying pathogenic variants independently of biomarker expression. Consequently, genomic newborn screening (gNBS) has the potential to detect additional conditions at presymptomatic stages, particularly those lacking early or detectable biochemical markers [19–21]. Moreover, gNBS has shown the ability to reduce false-positive rates and, in some cases, refine or modify initial biochemical diagnoses.

Studies using broader virtual gene panels further supported the diagnostic value of WES. NEXUS achieved diagnostic confirmation in 88% of IEM cases detected by tNBS and identified additional clinically actionable disorders not detectable by traditional screening (6.6%). In BabySeq, detection rates were 9.4% for childhood-onset genetic conditions and 3.5% for actionable adult-onset disease risks [14]. Notably, most newborns carried at least one recessive carrier variant, and a subset harbored variants potentially affecting drug metabolism, highlighting opportunities for genetic counseling and personalized therapeutic strategies. BabyDetect [15] demonstrated feasibility, high parental acceptance, and early detection of clinically relevant conditions beyond traditional NBS capabilities. Similarly, Shubina et al. [16] conducted one of the largest WES-based newborn screening pilots to date, screening 7,000 infants identifying treatable or preventable disorders, conditions of reduced penetrance, and chromosomal abnormalities, supporting the scalability of genomic newborn screening in clinical practice.

Despite these advances, the implementation of gNBS raises unresolved challenges, including interpretation of variants of uncertain significance (VUS), interpretation of findings in asymptomatic newborns definition of reporting criteria, management of uncertain or variable penetrance, and potential psychosocial impact on families [22,23]. Psychosocial assessments conducted in expanded NBS programs found no evidence of increased anxiety or psychological distress compared with standard screening [24,25]. Preliminary economic evaluations suggested that expanded screening strategies may be cost-effective by preventing advanced disease and reducing long-term healthcare costs [26].

Although long-term evidence remains limited, these studies underscore the potential clinical impact of genomic screening and support further evaluation of its integration into NBS programs [11–13,27]. This has led to additional pilot initiatives, including BeginNGS and ScreenPlus, with promising preliminary results [28,29]. BeginNGS demonstrated that early molecular diagnosis improved clinical intervention in over 41% of retrospectively evaluated cases, supporting the complementary use of genomic and biochemical screening [28,30].

In parallel with WES-based newborn genomics research, additional largescale initiatives are expanding the scope of newborn screening through complementary approaches. The GUARDIAN **study** [31] adopts a whole-genome sequencing (WGS) first approach to screen newborns for early onset, actionable genetic conditions; interim data from the first 4,000 infants show a **3.7% screen positive rate**, with the majority of these conditions not detectable by tNBS demonstrating the added diagnostic reach of genome sequencing in a highly diverse population. Complementing these efforts, **Begin**NGS proposes an international framework for newborn genome screening based on ultrarapid WGS, aiming to identify hundreds of treatable rare diseases at birth. As highlighted in recent analyses, BeginNGS [28] is positioned as a next generation model for universal genomic screening and as a platform to accelerate therapeutic development for rare disorders, reflecting growing momentum toward integrating genomewide testing into early life precision medicine. Collectively, these programs illustrate two parallel and increasingly convergent pathways in the evolution of newborn screening: expanded biochemical and targeted genetic testing (ScreenPlus), and largescale genome enabled screening (GUARDIAN and BeginNGS), both contributing to the broader shift toward combining genomic and biochemical approaches to enhance early diagnosis and intervention.

However, current reporting guidelines [32] restrict disclosure to pathogenic (P) or likely pathogenic (LP) variants, which may limit diagnostic resolution in cases where functional evidence is required for variant interpretation. In our population, approximately 20% of newborns with positive NBS results harbour at least one VUS (data not published), reinforcing the need for combined biochemical and genomic approaches.

In this context, untargeted metabolomics (UM) emerges as a powerful complementary tool. While tNBS covers approximately 50 conditions and gNBS could theoretically encompass over 300, UM provides functional insight into metabolic alterations and may aid in VUS reclassification [33]. UM captures broad metabolic profiles rather than predefined analytes, enabling both targeted and exploratory analyses. Its integration into routine diagnostics for inherited metabolic disorders in plasma and urine has already begun in the Netherlands, where metabolomic data are combined with genetic findings to improve diagnostic efficiency [34,35]. However, no prospective population-based study has yet evaluated the feasibility of integrating WES and UM in DBS within an established public NBS program.

Within this framework, the NBGenS project was designed as a prospective pilot study to evaluate the feasibility of a hybrid NBS model integrating tNBS, WES, and UM from the same DBS sample. NBGenS aims to enable early identification of severe, treatable pediatric diseases while generating evidence on gene selection, variant interpretation, clinical integration, cost-effectiveness, and the ethical, legal, and psychosocial implications of genomic newborn screening in a real-world healthcare setting in Spain.

## Methods

### Overview of study design

NBGenS is a prospective single-centre newborn genomic screening pilot study in Spain designed to expand tNBS. The implementation of the study will enable the early detection of additional conditions that currently are not detectable through tNBS but have available therapeutic options and may potentially be incorporated into routine clinical practice.

The study is based on a cohort of 550 newborns recruited at the University Clinical Hospital of Santiago de Compostela (Galicia, Spain) between December 2024 and September 2026, aiming to explore the impact of WES-based NBS as part of a pilot gNBS initiative and to evaluate outcomes for infants, families, and clinicians. This research was approved by the Santiago-Lugo Research Ethics Committee of Galicia (Spain) (registration code 2023/553) and conducted according to the revised Declaration of Helsinki.

The NBGenS Project team is a multidisciplinary consortium that combines clinical practice in neonatology, NBS, and clinical genetics with research capacity in bioinformatics, molecular genetics, and biochemistry. It also includes specialists in emerging therapeutic and nutritional strategies, and provides support in ethics and in the evaluation of psychosocial, behavioral, and health-related outcomes.

Fig 1 shows a diagram of the NBGENS Project workflow, which was designed to follow the American College of Medical Genetics and Genomics (ACMG) and ClinGen [36] recommendations [32] and align with the current best practices in the field [37]. The parents will receive detailed information and provide written informed consent before enrollment. If the parents decline to participate in the study, the reasons for decline will be collected.

**Fig 1:**
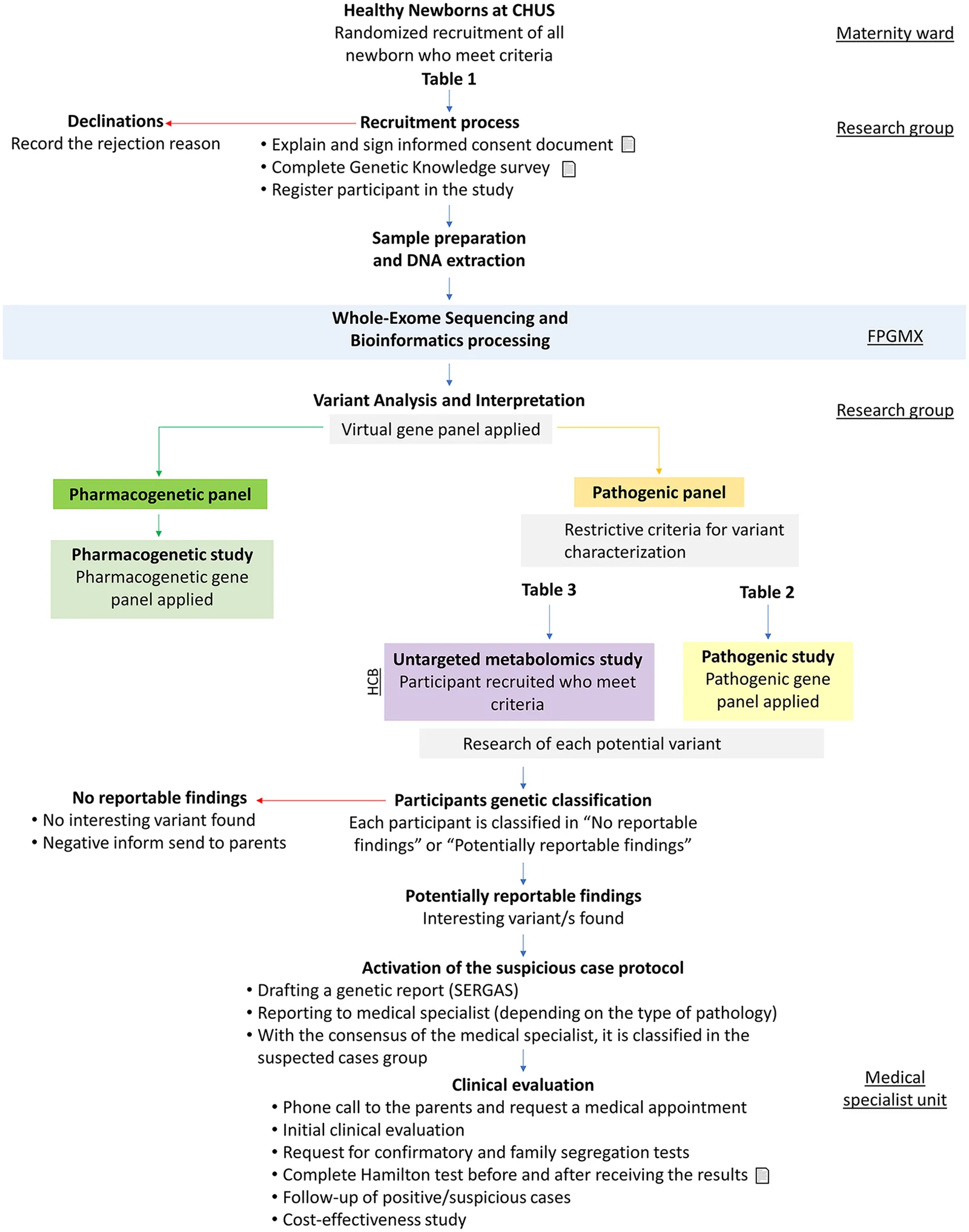
Overview of the NBGenS study design. Document icons indicate data collection points. Red arrows show where participants exit the workflow (parental refusal or absence of pathogenic findings). Underlined text indicates the team responsible for the processes shown at the corresponding level. CHUS: Complexo Hospitalario Universitario de Santiago; FPGMX: Fundación Pública Galega de Medicina Xenómica; HCB: Hospital Clínic de Barcelona; SERGAS: Servicio Galego de Saúde.

The DNA will be extracted from DBS samples obtained during routine tNBS and WES will be performed at Fundación Pública Galega de Medicina Xenómica (FPGMX) (Santiago de Compostela, Galicia, Spain). After sequencing, variants will be analyzed using two panels of genes, one targeting pathogenic or likely pathogenic variants and another assessing pharmacogenomic variants. An additional functional analysis using UM (Hospital Clinic de Barcelona, Barcelona, Spain) will be performed in those cases with selected VUS, LP, or P variants, as well as their combinations, depending on the inheritance pattern. The projected median time from recruitment to report delivery is 1 month, with a 90th percentile of 2 months.

In addition, the study will collect data to assess cost-effectiveness, ethical and social implications, and outcomes related to clinical practice and genetic counseling of implementing the additional screening. When a suspicious report is issued, the parents will complete a Hamilton Anxiety Rating Scale (HAM-A), using the validated Spanish version [38] to assess anxiety.

### Cohort

Based on actionable detection rates reported in previous studies [11,12] (7–10%; p = 0.06–0.09), and targeting a maximum margin of error of ±2.36% with a two-sided 95% confidence level (c = 2), we calculated a required sample size of 450 participants for NBGenS. To account for up to 10% potential attrition and to increase the precision of the study estimates through the inclusion of an additional 50 participants, recruitment was ultimately set at 550 neonates.

Enrollment will follow the predefined inclusion and exclusion criteria (Table 1). Participants will be randomly screened consecutively during the recruitment period.

**Table 1:** Inclusion and exclusion criteria for recruitment.

Inclusion criteria
• Parents aged ≥18 years who provide informed consent. • Neonates without a positive prenatal diagnosis of congenital malformations or chromosomal abnormalities.
**Exclusion criteria**
• Parents younger than 18 years. • Parents unwilling to complete study procedures. • Parents with cognitive or other impairments preventing informed consent. • Parents not consenting to their child’s participation. • Neonates not meeting the diagnostic criteria outlined above.

### Recruitment

The NBGenS research team will first assess newborns and their families to determine eligibility from the list provided by the maternity ward supervisor. Recruitment will begin with an explanation of the study procedures to the parents, and the provision of both the informed consent form and the genetic knowledge survey, allowing sufficient time for review and completion. This session will include an overview of study logistics, a genetic knowledge survey, a review of the types of reportable results, and a discussion of potential risks and benefits. Following the session, families will be guided through the different sections of both the informed consent document and the genetic knowledge survey and are given a reasonable amount of time to read, understand, and complete it at their own pace. Parents will be given contact information to address questions and to withdraw from participation at any time. Before collecting the signed forms, the case manager will review the genetic knowledge survey with the families to ensure that all sections have been appropriately completed. Incomplete, partially completed, or randomly completed genetic knowledge surveys will not constitute exclusion criteria. At this point, the newborn will be considered fully enrolled, and all study-related procedures will be initiated.

The structure of consent form consists of two sections. The first provides information on: the purpose of the document and the voluntary nature of participation; the study objectives; the reason why participation is offered to the newborn; details of participation (sample collection from the infant, analysis and communication of results, and data storage); potential discomforts or inconveniences; absence of direct benefits from participation; information to be obtained from the study; data-related information; financial disclosures; and contact information for the research team. The second section contains the operative elements of consent, including: the name of the project; a space for the name of the responsible party signing the consent; the specific points agreed to as part of consent; the management of collected data (deletion or retention after the study); and, finally, a designated space for the signature.

Consent will be obtained from both biological parents, when known, or from the legal guardians when applicable; however, only one parent’s or guardian’s information will be recorded in the consent document. In the case of twins or multiples, a separate consent form will be required for each newborn, although the genetic knowledge survey will be administered once per family. Families who do not return a complete informed consent form before hospital discharge will be considered passive withdrawals. Once the informed consent form has been signed, the newborn is considered fully enrolled in the study, and a copy of the signed document is provided to the family. The Spanish-language participant information sheet and informed consent form approved by the Research Ethics Committee, which will be used during recruitment, together with an English translation supplied solely for readers, are included in S1 File. Parents’ reasons for declining participation will be recorded at any stage of the enrolment process.

To evaluate parental understanding of genomic concepts and their comprehension of participation in the NBGenS project, the Genomic Knowledge Scale developed and validated by Langer et al. (2017) [39] will be used. The scale will be administered at the time of recruitment to assess parental genomic knowledge and to examine its relationship with participation. This instrument assesses baseline genomic literacy in the context of genomic sequencing and informed decision-making. The scale consists of 25 items organized into four conceptual domains: genes, genes and health, inherited family genes, and whole-exome sequencing. Each item is answered using a three-option format (“true,” “false,” or “don’t know”), allowing both correct knowledge and uncertainty to be captured. In addition to the knowledge items, basic demographic variables—including gender, age, and ethnicity—will be collected to contextualize responses and support subgroup analyses.

Questionnaire data will be analysed using descriptive statistics, with central tendency summarized as mean or median depending on data distribution. Results are graphically represented using interquartile range box plots. These analyses will be used to evaluate acceptance rates, identify factors associated with hesitation or refusal, and characterize concerns that may negatively influence willingness to participate.

A set of supporting materials will be used as part of the recruitment strategy to facilitate communication with families and enhance recruitment effectiveness. These materials will be used exclusively during the structured recruitment session to aid parental understanding prior to signing the informed consent form. i. Multilingual informational brochure: An informational brochure has been developed to provide a clear, visually structured overview of the project while maintaining scientific rigor. The first section introduces the context and relevance of neonatal genetic screening. The central section outlines the study procedures in a chronological and schematic format to improve comprehension of the workflow. The final section provides contact information and details on data protection and confidentiality. The brochure is available in Spanish, English, and Arabic. In addition, the recruitment team is proficient in Spanish, English, Galician, Catalan, and French, which will further support communication with families from diverse linguistic backgrounds (S2 Fig). ii. List of genes and conditions: A printed list containing 549 genes and associated disorders included in the project’s virtual panel wil be available during the session. Gene names and associated disorders were retrieved from Online Mendelian Inheritance in Man (OMIM) [40]. This resource will help convey the scope of the conditions screened and support clarification of any condition-specific questions raised by parents.

### Data collection

A standardized procedure will be used to collect participant information. Each participant will be assigned a unique study identification number in the order of recruitment for data pseudonymization. Turnaround times between each procedural phase will be systematically recorded for every participant. These will include the intervals from recruitment to DNA extraction, sequencing, variant interpretation, report generation, and result communication. Monitoring these timepoints will allow identification of potential bottlenecks within the workflow and facilitate optimization of process efficiency.

The Principal Investigator will print and archive the corresponding sheet under restricted access conditions; cases of declined participation will be documented in a separate tab of the same database.

Data from the source documents will be pseudonymized and recorded in a secure, controlled database within the Galician Health Service, stored on the hospital’s cloud infrastructure. Access will be restricted to authorized members of the research team, who will be able to consult essential participant identifiers, demographic information, study variables, newborn screening results, consent-related information, parental decisions regarding data use, and responses to the genetic knowledge survey. The database will be managed under the corresponding approved research line, with linkage codes accessible only to designated researchers. Genomic data and derived analytical files will be stored in pseudonymized form with restricted access for ten years from their generation. During the active phase of the project, they will remain in secure active storage and, after project completion, will be transferred to secure archival storage while remaining available for protocol-authorized reanalysis. At the end of the retention period, data will be securely deleted. This procedure will not affect validated clinical reports incorporated into the electronic health record, which will be retained in accordance with applicable regulations. Families will be informed of the retention period and planned data disposition during the consent process; no additional notification before deletion or anonymization is planned.

To assess parental anxiety throughout the diagnostic process, the HAM-A test [41] will be administered to both parents before receiving confirmatory results and again after disclosure. The clinician-rated HAM-A consists of 14 items scored on a 5-point scale, generating a total score from 0 to 56 that will reflect anxiety severity.

### Sample analysis and interpretation

#### Sample preparation.

Nurses perform the heel-prick test on all newborns between 24 and 72 hours after birth as part of the tNBS program. This involves collecting a small blood sample from the infant’s heel and depositing it onto DBS cards to fill the standard four circles, ensuring that the blood soaks through the paper completely. Once room temperature-dried, the DBS cards are transported to the Laboratory of Metabolic Diseases Unit (UDyTEMC) at the Clinical Hospital of Santiago de Compostela, following the tNBS workflow. No additional blood will be drawn for the study, as residual blood from the routine screening will be used, which will be sufficient for WES. The case manager will access the remaining sample using the internal code and will punch out three 3.2-mm discs using a biopsy punch. The discs will be placed in a 1.5 mL Eppendorf Safe-Lock DNA LoBind Tube (Eppendorf) and stored at 4°C until DNA extraction.

DNA will be extracted from DBS using the Chemagen kit (PerkinElmer). Briefly, samples will undergo lysis and protein digestion, followed by magnetic bead–based purification and elution in nuclease-free water. DNA concentration and purity will be quantified using both spectrophotometric (NanoDrop) and fluorometric (PicoGreen) assays. Extracted DNA will be stored at 4 °C when processing and shipment are scheduled on different days and will subsequently be delivered to the FPGMX genomic sequencing platform for WES.

An aliquot of the extracted DNA will be transferred to the genomic sequencing platform at FPGMX, where it will receive a unique internal identification code. Library preparation will be performed using the KAPA HyperPrep Kit, followed by target enrichment with KAPA HyperExome probes according to the manufacturer’s recommendations. Libraries will be sequenced on an Illumina NovaSeq 6000 system using 2 × 100 bp paired-end reads, achieving a mean coverage of 100–200× across coding regions and flanking intronic sequences. Bioinformatic processing will be conducted by FPGMX. Reads will be aligned to the human reference genome (hg38), and single-nucleotide variants (SNVs), insertions/deletions (indels), and copy number variants (CNVs) will be identified and annotated using established pipelines incorporating DRAGEN, Picard, mosdepth, bedtools, and Haplogrep. Variant interpretation will be restricted to genes included in the virtual NBGenS panel.

CNV candidates will be generated using the provider’s DRAGEN germline CNV pipeline (v4.2.7) [42], with copy-number calling enabled and panel-of-normals normalization, together with an in-house GATK-gCNV workflow based on a cohort model [43]. For GATK-gCNV, read-count intervals will correspond to the exome-capture targets and will be generated using PreprocessIntervals with a bin length of 0 and 250-bp padding. GATK-gCNV calls will be retained at a quality score ≥100 and DRAGEN calls at FILTER PASS. Two calls will be considered concordant, and therefore a high-confidence candidate, when they show ≥50% reciprocal overlap and affect the same gene in the same dosage direction (loss or gain); calls supported by a single caller will be reviewed but not classified as high-confidence. Calls larger than 3 Mb will be discarded as capture-driven artefacts, leaving an effective resolution down to a single captured target or exon.

The analytical performance of the combined workflow will be evaluated before clinical reporting to establish the validated minimum reportable range, particularly for single-exon events. CNV calling will be performed across the captured exome regions, but clinical interpretation and reporting will be restricted to genes included in the virtual panel. Off-panel CNVs will not be returned to families. Analytical performance will be estimated against reference materials with known copy-number states and previously array- or MLPA-confirmed in-house cases, reporting sensitivity and positive predictive value stratified by event size to define the minimum reportable range.

Panel CNVs will be classified according to the 2019 ACMG/ClinGen constitutional CNV framework [44], supported by ClassifyCNV [45], AnnotSV, and ClinGen dosage-sensitivity curations. Intragenic single- and multi-exon CNVs will be assessed according to gene dosage sensitivity and their predicted effect on the reading frame.

Concordant calls from both approaches will be considered high-confidence candidates. Clinically actionable CNVs will be confirmed using Multiplex Ligation-dependent Probe Amplification (MLPA) or chromosomal microarray analysis before communication to the family. The limitations of WES-based CNV detection, particularly for small, single-exon, intronic, or poorly captured events, will be considered when interpreting negative results.

A containment protocol will be applied in cases of technical issues. When sufficient material is available, the affected step will be repeated; if necessary, earlier stages of the protocol will also be revisited. Close coordination between the sequencing and research teams will be maintained to support turnaround times consistent with the projected schedule.

#### Gene panel.

The gene panel was selected according to the following criteria: i. gene–disease association (strong), ii. age of disease onset (<15 years), iii. effectiveness of early treatment (significance), and iv. severity and penetrance of the condition (severe and high).

Starting from an initial list compiled from the literature, existing panels, and other relevant sources, gene–disease pairs were classified into three groups: (i) pairs that adequately met all predefined inclusion criteria; (ii) pairs that met these criteria but required consideration of specific interpretative or clinical conditions, such as incomplete penetrance or variable expressivity; and (iii) pairs associated with conditions also detectable through conventional biochemical newborn screening. The detailed allocation of gene–disease pairs to these three groups is not shown in S3 Table, as this classification will remain subject to ongoing curation during subsequent panel updates. This classification will support documentation and periodic review of the panel but will not, by itself, determine whether a finding is reportable. A core panel was established from these groups and will be periodically updated as new evidence emerges, allowing gene–disease pairs to be added, removed, or reclassified. This dynamic-panel approach will ensure flexibility and scalability while preserving the scientific consistency and predefined inclusion standards of the panel (S3 Table). The current version comprises 549 genes and 603 gene–disease pairs.

#### Variant analysis and interpretation.

Variant interpretation and classification will follow the recommendations of the ACMG [32], the ACGS Best Practice Guidelines for Variant Classification in Rare Disease 2024 [46], and the Sequence Variant Interpretation (SVI) general recommendations for applying ACMG/AMP criteria [36].

Once sequencing has been successfully completed, the research team will be notified by the FPGMX to initiate the analytical process for the resulting variants using an internally developed FPGMX tool based on R Shiny and HPC scripting. This tool will allow all sequencing variants from a project to be consolidated, will keep their clinical and functional annotation up to date, and will enable rapid querying through a web interface that will communicate with the IGV genome viewer (referred to as the VarDB analysis platform).

To optimize variant interpretation, a set of restrictive criteria will be applied within the VarDB analysis platform to streamline the process (Table 2). Potential variants will be extracted for each participant and analyzed considering to their zygosity and the inheritance pattern of the disease selected in the panel. Although VUS may be observed, only pathogenic or likely pathogenic variants will be considered for subsequent analyses. VUS will be reserved solely for exploratory UM, which will be used as a functional assay to assess the pathogenicity of the variant.

**Table 2:** Restrictive criteria applied in VarDB for variant characterization.

Criteria	Value
InterVar_ACMG_verdict or ClinVar Significance	Likely pathogenic Pathogenic VUS*
AltCount	≥20
Quality	>8
Filter	PASS
vardb_Illumina_DRAGEN	≤ 750
MaxPopFreq	<0.05 (5%)
Annotation RefSeq	Delete intron_variant Intergenic_region Intragenic_variant Downstream_gene_variant Upstream_gene_variant

* VUS: Variants of uncertain significance will not be reported but will be reviewed for possible follow-up, future functional testing, and/or untargeted metabolomics analysis.

For variants associated with recessive diseases, the presence of additional variants in the same gene will be assessed to identify possible compound heterozygosity. In both scenarios -recessive variants (either in compound heterozygosity or homozygosity) and dominant variants (in heterozygosity or homozygosity)- only potentially reportable findings (PRF) will be considered. These findings will need to be supported by clinical evidence from major information sources, including Franklin [47], VarSome [48], OMIM [40], ClinVar [49], ClinGen [50], and PubMed [51].

Variant interpretation will be conducted in pairs by the research team, and results will be compared to reach a consensus conclusion. If variants of interest (referred to as *PRF*), are identified, the suspicious case protocol will be activated, and meetings will be convened with clinicians who are experts in the relevant disease category.

The VarDB analysis platform will use InterVar (2015) [52] to assign ACMG classifications to variants. Given this potential limitation, variant classifications will also be cross-checked against more up-to-date databases, such as Franklin.

A scheduled case reanalysis will be performed 12 months after the initial analysis. Thereafter, the virtual panel, gene–disease associations, interpretation resources, and relevant clinical recommendations will be reviewed annually throughout the data-retention period. Reanalysis may also be initiated at any time following new clinical, biochemical, metabolomic, or phenotypic information; a material update to the panel or bioinformatic workflow that may affect diagnostic sensitivity; new robust gene–disease or variant–disease evidence; new clinical recommendations; or reclassification of a previously identified variant with potential implications for diagnosis, prevention, surveillance, or treatment.

Families will be recontacted only when reanalysis identifies a new or modified result that meets the protocol reporting criteria and has potential clinical relevance. Reanalyses without clinically relevant changes will not be communicated individually, although their completion and outcome will be documented. For further details on the reanalysis triggers and family recontact criteria, please refer to S4 Fig.

Standard WES will not be considered reliable for the definitive detection of pathogenic repeat expansions. As a targeted complementary analysis, panel genes containing known pathogenic repeat regions will be screened using ExpansionHunter v5 and a curated variant catalogue applied to the WES reads. These calls will be treated as screening-level findings only. If clinical findings or family history suggest a repeat expansion disorder, such as Friedreich ataxia, fragile X syndrome, or myotonic dystrophy, an appropriate targeted assay, including repeat-primed PCR or Southern blotting, will be considered irrespective of the WES findings.

Mitochondrial DNA variants will only be assessed at positions meeting predefined depth and quality requirements because mitochondrial genome coverage from exon-capture WES will be variable. If mitochondrial disease is clinically suspected, dedicated mitochondrial DNA sequencing will be considered rather than relying on WES alone. Mitochondrial variants will be reported only at positions with a minimum read depth of 100× and base and mapping quality ≥20, and heteroplasmy will be quantified and reported at variant-allele fractions ≥10%.

Variants located near captured splice sites will be evaluated using SpliceAI and Pangolin with predefined thresholds. However, most deep intronic pathogenic variants will not be detected because they fall outside the captured regions. Genome sequencing or RNA-based testing may therefore be considered when clinical suspicion remains high despite a negative WES result. The predefined thresholds will be a SpliceAI or Pangolin delta score ≥0.20 as supporting evidence of a splicing effect, ≤ 0.10 as evidence against, and 0.10–0.20 as uninformative; for canonical splice-site positions without an exact delta score, a dbscSNV ada or rf score >0.6 will be used as a fallback.

Mosaic-sensitive tools, including DeepMosaic and MosaicForecast, will be used to support the identification of low variant-allele-fraction candidates. Nevertheless, low-level mosaic variants may remain below the detection threshold, and analysis of blood will not exclude tissue-restricted mosaicism. This analysis will target variant-allele fractions of approximately 1–30%, will be restricted to genes with neonatal-actionable mosaic phenotypes, and will require manual inspection of every candidate.

Repeat expansions, mitochondrial variants, deep intronic variants, and low-level mosaic variants will consequently remain underdetected by WES. Diagnostic yield estimates for these variant classes will therefore be interpreted as lower-bound estimates, and cases with strong clinical suspicion and negative or inconclusive WES findings may undergo appropriate targeted or orthogonal testing.

#### Genetic variants report.

Each case is individually reviewed by a multidisciplinary committee and discussed with the responsible clinician before disclosure, after which a report is prepared and issued by clinical molecular geneticists from the Laboratory of Metabolic Diseases Unit. The structure and content of reports are adapted from genomic reports routinely developed following the ACMG recommendations [32]. These reports consist of a single document: the first page includes the delivery address of the family residence, participant details, a “Results Summary” outlining the findings, followed by an “Interpretation Summary,” and a final “Summary of the Type of Analysis Performed.” The second page includes Annex I (list of genes analyzed in the NBGenS virtual panel) and Annex II (gene coverage specifications).

Following the completion of the analytical and interpretative process for each participant’s variants, cases are classified into one of two possible groups: *No reportable findings* (NRF) or *Potentially reportable findings* or PRF. Classification as PRF requires the identification of variants that meet the criteria for reporting-namely, pathogenic, or likely pathogenic variants in genes supported by scientific evidence demonstrating pediatric-onset disease causality for which early intervention is possible in a zygosity compatible with the inheritance pattern of the disease. For conditions showing variable expressivity, reporting decisions are based primarily on the evidence available for the specific variant and, when this is insufficient, on the corresponding genotype and gene–disease association. All other scenarios are classified as NRF, including the absence of reportable pathogenic or likely pathogenic variants; VUS, even when selected for untargeted metabolomics or subsequent reevaluation; isolated heterozygous pathogenic or likely pathogenic variants in genes associated with autosomal recessive diseases; and pathogenic or likely pathogenic variants associated with low, incomplete, variable, or insufficiently established penetrance, unless they are considered clinically relevant and enable preventive, surveillance, follow-up, or therapeutic measures with potential benefit for the child. These findings will not be communicated as clinically positive results, although they may be retained for subsequent research evaluation.

ACMG secondary findings are neither actively sought nor reported. Variant filtering, interpretation, and reporting are restricted to the predefined NBGenS disease and pharmacogenetic panels; variants outside these analytical scopes are excluded from review and therefore cannot be returned as secondary findings within the NBGenS workflow. Any subsequent analysis beyond the original panel prompted by the development of a relevant phenotype will be considered a separate clinically indicated investigation and managed according to the applicable clinical, ethical, consent, and ACMG secondary-findings procedures. For further details on the variant interpretation and reporting decision framework, please refer to S4 Fig.

For participants classified as NRF, reports are sent by regular postal mail following standard tNBS procedures. For suspicious cases with a PRF, a specific protocol is activated, and the report includes an additional summary of the variant of interest. These reports are never mailed directly to the family. Instead, each case is reviewed in interdisciplinary meetings with the relevant clinical specialists, after which parents are informed of the findings and invited to an in-person consultation with pediatricians and clinical geneticists to receive the results, genetic counseling, and appropriate follow-up. All parent-directed communications (phone calls and in-person visits) follow the best practices described by Remec et al. [20].

To streamline report generation, a Visual Basic for Applications (VBA)-based macro is employed for automation. The initial Visual Basic for Applications code is developed with the aid of artificial intelligence but undergoes review and refinement by expert bioinformaticians (S5 File).

### Clinical evaluation and follow-up

For participants with PRF, an initial clinical evaluation will be performed. This visit will take place in the presence of a pediatrician specialized in the participant’s disease category, a clinical geneticist, and the case manager, who will accompany families throughout all steps of the study. During this visit, the current health status of the participant will be assessed through physical examination and clinical questioning, and the identified genetic findings will be explained along with their potential clinical implications. Family history will be explored to identify possible segregation patterns, and genetic testing of the parents will be requested to investigate the inheritance of the detected variant(s). When necessary, additional targeted tests may be ordered to further evaluate the health status of both the participant and the parents. Ample time will be provided for parents to express questions or concerns, acknowledging that these consultations may involve complex and emotionally challenging information. At the end of the visit, families will receive a written summary of the discussion, the genetic report, and the Hamilton Anxiety Scale to assess stress or anxiety levels.

Follow-up will be conducted for all newborns with a positive genetic finding at predefined time points: day 0, day 15, day 30, day 60, and day 90. The objective will be to monitor the onset of clinical symptoms, evaluate the need for treatment, and collect data to complete the cost-effectiveness analysis.

### Primary expected outcomes

The study will establish and evaluate a pioneering multi-step clinical workflow for delivering complete genomic sequencing of newborns in a screening format. The development of this workflow will encompass: 1. periodic review of the virtual gene panel, 2. protocol development, 3. recruitment and registration, 4. sample preparation and DNA extraction, 5. genomic sequencing, 6. sequencing data analysis and interpretation, 7. participant classification into reporting groups, 8. report generation, 9. communication of results to participants and healthcare professionals, 10. follow-up period, and 11. integration of information into clinical records. The development and implementation of an efficient workflow will provide crucial insights into what processes are effective and which barriers exist. To analyse this workflow, the time intervals between each step will be recorded.

Additional outcomes will include: the demographic distribution of parents who choose to enrol in a gNBS study; factors that may influence participation and confidence, based on the questionnaires completed by the parents; the process and rationale for selecting genes and variants for reporting, including which findings will be included or excluded and which categories of information (e.g., variants with limited or uncertain evidence), will not be returned but may warrant reconsideration; evaluation of optimal formats for reporting genomic results, providing genetic counselling, and conducting clinical follow-up; the contribution of genomic data to the early medical management of infants beyond standard NBS; the identification of hidden or subclinical biomarkers or phenotypes in infants carrying risk variants, including manifestations detectable only through UM; and the medical, behavioural, and economic impact of integrating genomic sequencing of genes associated with treatable neonatal diseases or drug metabolism into the care of apparently healthy, at-risk, or ill newborns.

### Secondary outcomes

#### Pharmacogenetic study.

The pharmacogenetic study will constitute a secondary objective of the NBGenS project. It will aim to determine the detection rate of variants in genes with strong pharmacological associations relevant to current or potential paediatric treatments and to assess their possible inclusion in a gNBS programme. Compared with the primary objective, this analysis will differ in the selection of genes, the variant analysis and interpretation strategy, and the reporting of results.

The initial gene–drug list will be compiled from PharmVar [53], the Centers for Disease Control and Prevention (CDC) Tier 1 genomic applications [54], the AEMPS Pharmacogenomic Biomarker Database [55], and PharmGKB databases [56]. The resulting research universe will comprise 1,160 gene–drug associations involving 81 genes and 464 drugs, as detailed in S6 Table.

Star alleles will be inferred from aligned WES CRAM files using PyPGx v0.26 for the pharmacogenes supported by the software. Statistical haplotype phasing will be combined with copy-number estimation based on normalised read depth for CNV-defined loci, including *CYP2D6* and *UGT1A1*. Genes not resolved using PyPGx will be diplotyped from the joint-genotyped VCF using PharmCAT v3.2.0, while HLA-B*57:01 and HLA-A*31:01 will be typed using HLA-LA.

Pharmacogenetic findings will be organised into clinical and exploratory tiers. The clinical tier will be restricted to CPIC Level A actionable gene–drug pairs involving 22 genes for which prescribing guidance from CPIC, DPWG, or the FDA is available. Within this tier, priority will be given to associations with PharmGKB evidence levels 1A–2A and to AEMPS-authorised medicines with paediatric relevance. Associations with lower levels of evidence, without paediatric relevance, or involving medicines not covered by the AEMPS criteria will be retained for possible future reanalysis but will not be returned under this protocol.

The recognised limitations of exon-capture WES at complex pharmacogenetic loci will be considered during interpretation. In particular, WES may not reliably resolve *CYP2D6*–*CYP2D7* hybrid alleles, whole-gene deletions or duplications, or pseudogene interference, and rare *CYP2C19* structural variants may be missed. If a reliable *CYP2D6* copy-number or structural call cannot be obtained, no clinically actionable structural result will be issued from WES data alone.

All clinically actionable pharmacogenetic findings will undergo orthogonal confirmation using an appropriate targeted method, such as a TaqMan copy-number or genotyping assay, long-range PCR, Sanger sequencing, or a validated pharmacogenetic genotyping panel, before communication. A healthcare professional with training in pharmacogenetics will communicate these findings to families through a separate report, which will be incorporated into the electronic health record of the Galician Health Service. Findings with insufficient clinical relevance, analytical reliability, or actionability will not be returned.

This secondary analysis will highlight the considerable versatility and potential utility of the genomic information that will be collected at the individual level, enabling both alternative analytical approaches and future reanalysis that may prove clinically valuable should new indications arise during the study period.

#### Untargeted metabolomics study.

After completing tNBS tests and WES, variants will be analyzed according to the previously described criteria (Table 2) using the VarDB analysis platform. Because available resources and analytical capacity do not permit untargeted metabolomic evaluation of every VUS, variants will be prioritized for this analysis. VUS prioritized for untargeted metabolomics are defined as variants that, following initial classification according to ACMG/AMP and ClinGen recommendations, could potentially reach the likely pathogenic category if metabolomic findings provided additional applicable phenotypic or functional evidence, such as PP4 or PS3. The analytical criteria that will be used to determine participant eligibility for UM analysis are summarized in Table 3.

**Table 3:** Inclusion and exclusion criteria for NBGenS participants in untargeted metabolomics.

Inclusion criteria
• Genes associated with autosomal dominant diseases: participants carrying one or more LP, P, or VUS prioritized for untargeted metabolomics, either in the heterozygous or homozygous state. • Genes associated with autosomal recessive diseases: participants carrying two or more LP, P, or VUS prioritized for untargeted metabolomics in either the homozygous or heterozygous state (including cases with a single homozygous variant). • Genes associated with X-linked diseases: male participants (XY) carrying one or more variants in hemizygosis, or female participants (XX) carrying two or more LP, P, or VUS variants in either homozygosis or heterozygosis (including cases with a single homozygous variant).
**Exclusion criteria**
• Participants who have signed version 1.0 of the informed consent, which does not include untargeted metabolomics. • Absence of LP, P, or VUS variants. • Presence of LP, P, or VUS variants that do not meet the inclusion criteria (e.g., carriers).

LP: Likely pathogenic; P: Pathogenic; VUS: Variants of uncertain significance

Participants meeting any of the inclusion criteria will have their samples prepared for shipment to the designated reference laboratory (Hospital Clinic de Barcelona, Spain). To preserve pseudonymization, all samples will be identified exclusively by their NBGenS study identification code.

Correctly labelled samples will be organized using a standardized system of envelopes and protective bags before shipment. Metabolites will be extracted from the DBS samples using a water/methanol solution. After evaporation of the eluate, the residue will be reconstituted with water containing 0.1% formic acid and injected into a Shimadzu LC40 ultra-performance liquid chromatography (UPLC) system coupled to a high-resolution time-of-flight mass spectrometer (X500R-QTOF, Sciex S.A). Chromatographic separation will be performed using an Acquity HSS T3 column (2.1 × 100 mm, 1.8 μm; Waters, Milford, MA, USA).

Data analysis will be performed using SCIEX OS software in combination with an in-house–developed automated bioinformatic pipeline. Metabolite levels in each participant will be compared with those observed in a reference control cohort using Z-scores, and values ≥ +2 or ≤ −2 will be considered potentially abnormal. The pipeline will perform data processing, Z-score calculation, and the initial identification of altered metabolites. All metabolomic profiles will subsequently be reviewed by a multidisciplinary team with expertise in clinical metabolomics and inherited metabolic disorders. This review will consider potential confounding factors, including prematurity, gestational age, nutritional status, concomitant medical treatments, intercurrent illness, and metabolic patterns previously described in these settings. Only abnormalities consistent with the pathophysiological mechanism of the disorder under evaluation and not reasonably explained by these potential confounders will be considered supportive evidence for clinical interpretation. The reference control cohort will comprise unaffected newborns analysed on the same platform and matched for gestational and postnatal age, feeding, and analytical batch. Because a |Z| ≥ 2 threshold will flag a proportion of features by chance across the untargeted profile, an isolated Z-score will not be treated as evidence in itself: only alterations that are biologically consistent with the candidate variant and reproducible will be retained, and no metabolite will contribute as ACMG evidence on the basis of the Z-score alone.

The data obtained will be systematically recorded in a Microsoft Excel database. Prior to metabolomic analysis, the dataset will include each participant´s unique study identification code and variants of interest, including the gene, OMIM entry, inheritance pattern, zygosity, NM accession, variant type, ACMG classification, specific comments, and timestamps for the different procedural stages.

Once UM results are available, the following information will be documented: metabolomics outcome (normal, abnormal, or insufficient sample), metabolite name and type of alteration (increase or decrease), potential correlation between the biochemical alteration and the identified variant, current ACMG classification, and any proposed reclassification.

This complementary approach will enable the identification of biochemical abnormalities associated with variants of uncertain or incompletely characterized clinical significance and will provide additional evidence for their interpretation. In cases where a VUS is identified together with an untargeted metabolomic profile compatible with the disorder under evaluation, the metabolomic finding will be considered an additional source of evidence within the structured ACMG/AMP classification framework but will not, in isolation, determine variant reclassification or clinical reporting. Its applicability as phenotypic or functional evidence—for example, PP4 or PS3, respectively—and the strength assigned will be determined according to the disease mechanism, the specificity of the metabolic profile, and the degree of analytical and clinical validation of the assay, in accordance with ACMG/AMP and ClinGen recommendations. Each case will be reviewed by a multidisciplinary committee that will integrate the metabolomic findings with population, computational, segregation, phenotypic, functional, and case-level evidence. A variant will be reclassified as likely pathogenic and clinically communicated only when the combined independent evidence meets one of the ACMG/AMP combinations established for that category, no relevant contradictory evidence is present, and the finding meets the reporting criteria defined in the protocol. Variants that do not meet this threshold will remain classified as VUS, will not be communicated as clinically positive findings, and may undergo follow-up or further analysis exclusively within the research context.

In addition, UM will allow the exploration of a much wider range of biochemical alterations than tNBS, enabling the detection of metabolic disturbances linked to a broader spectrum of diseases. It will also offer a broad range of measurable biochemical features that may be leveraged for biomarker discovery.

Sequencing the entire exome will provide an additional advantage: when a metabolic alteration is detected that does not correlate with previously identified variants, the exome may be retrospectively interrogated to perform a targeted search across all known genes potentially implicated in the observed biochemical abnormality. These integrated findings may support the formulation of hypotheses beyond tNBS and provide deeper insight into individualized risk assessment and metabolic characterization. However, the integration of these approaches will also represent a significant methodological and implementation challenge; therefore, their added clinical value, feasibility within the screening framework, and optimal strategies for communicating results will need to be carefully evaluated.

#### Cost-effectiveness study.

A Markov decision-analytic model will be developed to perform both cost-effectiveness and cost-utility analyses, estimating the discounted incremental cost per life-year gained and per quality-adjusted life-year (QALY) gained for neonatal genetic screening compared with no screening. The analysis of the economic implications of gNBS with particular emphasis on cases in which a PRF is identified and classified as *suspicious* following clinical consensus, will incorporate key parameters including disease prevalence, variant allele frequency, degree of associated disability, diagnostic test reliability, comparisons between early and delayed intervention, the preventive potential of early detection, available therapeutic options, and the potential benefits for relatives in whom familial segregation may be identified.

Estimates of the expected net changes in costs and life expectancy derived from genetic screening will be used to calculate incremental cost-effectiveness ratios. Sensitivity analyses of the main variables will be performed using Monte Carlo simulations. The economic evaluation will apply discount rates to both costs and health outcomes, with a base case of 3% and a range of 0% to 5% for sensitivity analyses. In addition to the cost-effectiveness analysis, a budget impact analysis will be conducted, given the need to estimate the financial consequences of adopting or expanding a new healthcare technology or intervention within a specific context [57].

### Statistical methods

The detection rate will be established based on the results of the genetic panels analysed. The frequency of *de novo* variants, variants associated with recessive inheritance, and copy number variants will also be estimated.

Differences in paired proportions will be evaluated using McNemar’s test. Incremental cost-effectiveness ratios will be calculated through sensitivity analyses of key variables using Monte Carlo simulations. For the economic evaluation, discount rates will be applied according to the formula *PV = FV/(1 + Dr)*^*t*^, where *PV* represents the present value, *FV* the future value, *Dr* the discount rate, and *t* the elapsed time. A base discount rate of 3% will be applied to both costs and health outcomes, with a range of 0% to 5% considered in sensitivity analyses.

### Timeline

The study is planned to finish recruitment in the second quarter of 2026, and it is anticipated to be completed in the first quarter of 2027.

#### AI-assisted language editing.

During the preparation of this manuscript, the authors used ChatGPT (OpenAI) to assist with language editing and improvement of the text and to generate the non-data-bearing illustrative images used in the design of S2 Fig, based on prompts written by the authors. The tool was not used for data analysis, interpretation, or generation of scientific content. All generated content was carefully reviewed and edited by the authors, who take full responsibility for the final version of the manuscript.

## Discussion

The NBGenS project is a pilot study on the implementation of genomic medicine in newborns. It will use a design intended to address some of the concerns raised in this field regarding the potential psychological impacts of presenting genomic information to the families of newborns. Given its complexity, the initial design and implementation phases have provided valuable insights not only into the clinical and ethical dimensions of neonatal genomic screening, but also into the practical and organizational challenges inherent to its integration within routine care.

During an initial phase of recruitment, in which a limited number of participants were enrolled, one of the first logistical challenges identified was the restricted enrolment window, as healthy newborns are typically discharged within 48 hours of birth. This narrow timeframe provided parents -already fully engaged in the immediate care of their newborn- little opportunity to reflect on participation or complete the study survey. Moreover, the requirement for informed consent from both parents meant that the absence of one parent during hospitalization often hindered discussions and resulted in non-participation. This limitation has already been reported in previous studies [13,58,59]. To mitigate these constraints, the protocol was adapted to allow consent after hospital discharge, without a fixed time limit, thereby offering families a less stressful environment in which to make an informed decision. Nevertheless, as suggested in previous studies [60,61], prenatal recruitment may represent a valuable strategy to improve participation and provide families with additional time for reflection in future studies.

During protocol development, outcome assessment required careful calibration to balance comprehensive evaluation of parental experiences with the need to minimize respondent burden. To avoid overwhelming families during a sensitive postnatal period, the data-collection instruments were deliberately streamlined, and the number of questionnaires administered was limited.

Additional considerations will include potential unequal sex representation and language-related barriers. Late-stage recruitment monitoring will be used to promote balanced sex representation. Furthermore, while the ethics-approved informed consent form are available only in Spanish, multilingual informational materials and tailored communication strategies is implemented to support comprehension. Enrolment is restricted to families with sufficient Spanish and/or English literacy to ensure full understanding of the consent process. Linguistic adaptation is necessary, as already considered in previous studies [62,63], mainly due to the increasing proportion of immigrants in the Spanish population, who will ultimately become part of the country’s genetic background [64–66].

As genomic sequencing becomes increasingly integrated into clinical care, its incorporation into universal NBS is emerging as a realistic possibility. The NBGenS Project will aim to generate objective evidence on the risks and benefits of neonatal genomic sequencing, including its impact on child health, psychosocial outcomes for families, and the clinical utility of genomic information for healthcare professionals. At the same time, evaluating real-world implementation will inevitably bring into focus the methodological and interpretative challenges inherent to screening asymptomatic populations.

One of the principal limitations of genetic screening in this context will be the frequent detection of VUS [67] or theoretically predicted pathogenic or likely pathogenic variants supported solely by bioinformatic evidence, in the absence of previously reported clinical cases. Such findings will not be disclosed because of insufficient evidence of pathogenicity; however, they will remain subject to ongoing reanalysis. If reclassification to pathogenic or likely pathogenic occurs, or if a participant develops clinical features consistent with a genetic disorder, these variants will be carefully re-evaluated and reported with appropriate genetic counseling and consent. Currently, comprehensive reanalysis requires substantial manual effort and time, highlighting the potential value of automated and AI-assisted tools to support evidence retrieval, variant prioritization, and standardization of the reanalysis process, while final classification and reporting decisions will remain subject to expert multidisciplinary review.

In this regard, the integration of UM with genomic and conventional biochemical screening will provide an important complementary advantage [68,69], offering biochemical evidence that may help refine variant interpretation and reduce uncertainty. ScreenPlus [70] illustrates the value of multitiered strategies combining biochemical assays with targeted molecular testing. However, BabyDetect [15] represents a closer methodological comparator for NBGenS, as both are prospective, single-centre studies embedded in a public maternity setting and evaluate genomic screening as a first-tier approach for severe, actionable paediatric disorders. BabyDetect demonstrated high population acceptance and identified clinically relevant conditions that had not been detected through conventional NBS, thereby providing empirical support for the added diagnostic reach of genomic screening.

The BabyDetect experience also illustrates several methodological trade-offs that are directly relevant to NBGenS. Its targeted sequencing strategy relied on a predefined capture panel and on automated filtering of previously curated pathogenic or likely pathogenic variants, followed by expert review. Although this approach substantially reduced the number of variants requiring manual assessment, a previously unreported pathogenic *TJP2* variant was not initially flagged, and modification of the targeted panel required a new analytical validation process. In comparison, NBGenS will use WES with a dynamically curated virtual panel, allowing the analysed gene set to be updated without redesigning the sequencing assay, while retaining expert multidisciplinary review of potentially reportable findings. NBGenS will also extend the implementation framework by using residual DBS collected through routine NBS and by incorporating selected UM analyses, pharmacogenetics, psychosocial assessment, clinical follow-up, and health-economic evaluation. These differences will allow the study to examine whether a broader and more adaptable workflow can be integrated into an established public NBS programme while maintaining appropriate interpretative and reporting safeguards.

Furthermore, rigorous gene curation represents a critical component of responsible genomic screening. Prior to commencing enrolment, more than 1000 genes were reviewed for disease association strength, inheritance pattern, age of onset, and penetrance, of which approximately 600 met the criteria for return within NBGenS. As new gene–disease evidence continues to emerge, the panel will be dynamically updated, allowing previously excluded genes to become eligible for reporting and vice versa. Although this curated reference list is expected to substantially reduce interpretation time, the manual nature of the review process will carry a risk of obsolescence [71,72]. Future development of AI-supported systems for continuous evidence aggregation and panel updating, as demonstrated in other similar initiatives [73,74], may represent a sustainable decision support for expert curation, while final inclusion, exclusion, and reporting decisions will remain subject to multidisciplinary review.

Additional methodological considerations relate to analytical validity, particularly the potential limitations of current sequencing approaches in detecting pathogenic variants located in challenging genomic regions, such as deep intronic insertions or deletions. Although WES provides broad coverage of coding regions and captures the majority of known pathogenic variants associated with monogenic disorders, WGS may offer a more comprehensive assessment enabling the detection of deep intronic variants, structural variants, mitochondrial changes, repeat expansions, or methylation disorders—categories of pathogenic variation that are increasingly relevant to pediatric onset genetic diseases. Reviews of genomic newborn screening underscore that WGS offers more uniform coverage, improved detection of copy number variations, and the ability to reanalyze genomic data as new gene–disease associations emerge. On the other hand, WGS introduces challenges including higher costs, increased complexity of variant interpretation, greater incidence of uncertain findings, and more substantial ethical considerations regarding secondary or adult-onset findings.

Turnaround time will also represent a critical operational parameter. During the initial recruitment phase, the interval between recruitment and either report issuance for participants with no reportable findings -or direct communication in cases classified as suspicious- was estimated at approximately one month. Upon study completion, each procedural phase will be systematically reviewed to identify bottlenecks and opportunities for optimization, with particular attention to communication interfaces among sequencing laboratories, research staff, clinicians, and families. Strengthening coordination and establishing standardized protocols will be essential to ensuring efficiency in future large-scale implementation.

Finally, this study will also provide preliminary evidence on the economic impact and healthcare burden associated with neonatal genomic sequencing within the national health system. We anticipate that the findings from the NBGenS Project will serve as an evidence base for policy decisions related to the potential adoption of universal genomic newborn screening.

## Supporting information

S1 File Participant information sheet and informed consent form used in the NBGenS study, version 2. (PDF)

S2 Fig Multilingual informational brochure used during recruitment. (PDF)

S3 Table NBGenS virtual gene–disease panel, version 4.1. (XLSX)

S4 Fig Decision algorithm for variant interpretation, reporting, reanalysis, and recontact in the NBGenS protocol. (PDF)

S5 File Visual Basic for Applications code used to assist report generation. (PDF)

S6 Table Pharmacogenetic gene panel included in the secondary analysis. (XLSX)

## Abbreviations

ACMG: American College of Medical Genetics and Genomics
CDC: Centers for Disease Control and Prevention
CNV: Copy Number Variant
DBS: Dried Blood Spot
Dr: Discount rate
FPGMX: Fundación Pública Galega de Medicina Xenómica
FV: Future Value
gNBS: genomic newborn screening
HAM-A: Hamilton Anxiety Rating Scale
hg38: Human Reference Genome
IEM: Inborn Errors of Metabolism
indels: insertions/deletions
LP: Likely pathogenic variant
NBS: Newborn Screening
NHGRI: National Human Genome Research Institute
NICHD: Eunice Kennedy Shriver National Institute of Child Health and Human Development
NRF: No reportable findings
NSIGHT: Newborn Sequencing in Genomic Medicine and Public Health
OMIM: Online Mendelian Inheritance in Man
P: Pathogenic variant
PRF: Potentially reportable findings
PV: Present Value
QALY: Quality-Adjusted Life-Year
SNV: Single-Nucleotide Variant
t: Elapsed time
tNBS: Traditional Newborn Screening
UDyTEMC: Laboratory of Metabolic Diseases Unit
UM: Untargeted Metabolomics
UPLC: Ultra-Performance Liquid Chromatography
VarDB: FPGMX variant analysis platform
VBA: Visual Basic for Applications
VUS: Variant of Uncertain Significance
WES: Whole Exome Sequencing
WGS: Whole-Genome Sequencing
