# Supplementary material for: The NBGENS project protocol: Pilot study for genetic newborn screening in Galicia
Source: PLoS One. 2026 Oct 8;21(10):e0359270. doi: 10.1371/journal.pone.0359270 (PMC13649029; doi:10.1371/journal.pone.0359270)
Supplement: Supplementary file 1 [file pone.0359270.s001.pdf]

HOJA DE INFORMACIÓN PARA PADRES Y MADRES/ TUTORES LEGALES DE MENORES PARTICIPANTES

TÍTULO DEL ESTUDIO: Estudio piloto de cribado genético neonatal: NBGenS

INVESTIGADOR María Luz Couce Pico
CENTRO: Hospital Clínico Universitario de Santiago de Compostela

Este documento tiene por objeto facilitarles información sobre un **estudio de investigación** en el que se invita a su hijo y a ustedes a participar. Este estudio fue aprobado por el Comité de Ética de la Investigación de Santiago/Lugo.

Si deciden participar en el mismo, deben recibir información personalizada del investigador, **leer antes este documento** y hacer todas las preguntas que precisen para comprender los detalles sobre el mismo. Si así lo desean pueden llevar el documento, consultarlo con otras personas y tomarse el tiempo necesario para decidir si participar o no.

La participación en este estudio es completamente **voluntaria**. Ud. pueden decidir no participar o, si aceptan hacerlo, cambiar de parecer retirando el consentimiento en cualquier momento sin tener que dar explicaciones. Le aseguramos que esta decisión no afectará a su relación con los profesionales sanitarios que le atienden ni a la asistencia sanitaria a la que Ud. tienen derecho.

¿Cuál es la finalidad del estudio?

El cribado neonatal es un programa de salud pública que garantiza la detección de algunas enfermedades congénitas (7-34 enfermedades en España) tratables en los recién nacidos permitiendo una detección temprana antes de que se presenten síntomas graves. La finalidad de este estudio es implementar un cribado neonatal ampliado, que incluye el análisis bioquímico y genético, con el fin de detectar más de 300 enfermedades de inicio pediátrico y de baja prevalencia, para las que se dispone de tratamiento/intervención precoz. Complementado, además, con una análisis metabolómico en la misma muestra, que abre la posibilidad de identificar nuevos marcadores bioquímicos para las enfermedades cribadas.

¿Por qué ofrecen participar a mi hijo?

Invitamos a participar a su hijo y a ustedes, sus progenitores, porque cumplen los criterios de selección para realizar un cribado genético neonatal y se encuentran en el servicio de Obstetricia del Complejo Hospitalario Universitario de Santiago de Compostela.

¿En qué consiste su participación?

La participación de su bebé en este estudio consiste en:

- □ recogida de sus datos clínicos al nacimiento,
- □ a partir de la muestra de sangre en papel, se realizará el análisis metabolómico y el análisis genético (previa extracción de ADN).

La participación de los progenitores en este estudio consiste en:

- □ recogida de sus datos clínicos e historia familiar,
- □ realización de dos cuestionarios, uno de valoración de conocimiento de genética y otro de valoración del estado de ansiedad, en dos ocasiones:
    i. Al inicio, tras el consentimiento de su participación y la de su bebé en este estudio
    ii. Tras la emisión del informe final con el resultado del cribado.

- ¿Cuándo y cómo se obtendrán las muestras de su bebé?

Las muestras de sangre se obtendrán según la práctica clínica habitual, a partir de las 24h-48h de vida del bebé, en papel coincidiendo con la recogida de muestra para el cribado neonatal convencional, sin ser necesaria una extracción adicional. La extracción de sangre consiste en un pinchazo en el talón del bebé, realizado por personal sanitario, en donde se impregna la sangre en un papel absorbente homologado. Esta muestra, por protocolo de práctica clínica habitual, se envía a la Unidad de Metabolopatias del Hospital Clínico Universitario de Santiago de Compostela. A partir de la sangre seca recogida en el papel absorbente, se hará por un lado i) el análisis bioquímico para el cribado neonatal convencional, incluida en la asistencia habitual, y, además, ii) la extracción del ADN para el estudio genético, iii) análisis metabolómico no dirigido.

En alguna situación se podría necesitar nueva muestra por si la calidad de ADN extraída de la muestra de sangre no es suficiente para garantizar una buena interpretación de los resultados.

- ¿Qué se va a analizar?

En ambos análisis, bioquímico y genético, se pretende detectar enfermedades que son de **baja prevalencia**, de inicio pediátrico/infantil y que tienen **tratamientos disponibles** para mejorar el pronóstico del desarrollo de su bebé.

El análisis bioquímico para el cribado neonatal convencional va a permitir la detección de 34 enfermedades metabólicas (https://www.sergas.es/Saude-publica/Patoloxias-incluidas-programa-galego-xeneticas).

A partir del ADN extraído, se va a proceder a la secuenciación del exoma completo de su bebé para el análisis genético. Este análisis implica el estudio de dos grupos de genes previamente seleccionados:

1. Grupo PedAC, que incluye genes asociados a enfermedades pediátricas y que cumplen unos criterios específicos de selección: son asociados a patologías conocidas y bien descritas, con tratamiento disponible y que mejoran el pronóstico del niño/a afectado/a. Este análisis pretende proporcionar por un lado la confirmación del resultado bioquímico, y por otro, ampliar la detección de hasta cerca de 300 enfermedades (cardiacas, endocrinología, hemoglobinopatías, inmunodeficiencia, neurológicas, neuromuscular, metabólicas, sordera).
2. Grupo Farmacogenética, que incluye genes asociados a una respuesta terapéutica individual. Este análisis pretende identificar aquellos genes que pueden afectar la forma como su hijo/a responde a ciertos medicamentos. Esta información podría ayudar a elegir qué medicamentos o dosis de medicamentos pueden ser más seguras y adecuadas para su hijo/a, aumentando su eficacia y disminuyendo las posibles reacciones adversas.

- ¿Cómo se informarán los resultados?

Los resultados del cribado bioquímico convencional, seguirá las pautas de la asistencia habitual donde los resultados son informados en el plazo de una semana. La familia recibirá los resultados del estudio genético por carta en el domicilio que haya facilitado, al cabo de 4 semanas aproximadamente. Si pasado este plazo no se han recibido, usted puede comunicar la incidencia al Laboratorio de Metabolopatías, llamando al teléfono 981 950 100, de lunes a viernes, en horario de 8:30 a 14:30. Cualquier otra duda respecto al estudio piloto debe consultarla con su pediatra.

Los resultados del informe genético objecto de estudio de este trabajo pueden ser los siguientes:

- □ Negativo + Farmacogenética
- □ Positivo + Farmacogenética

El plan de seguimiento según resultado del informe se describe a continuación:

- □ Resultado negativo: no son detectadas alteraciones tanto en el análisis bioquímico como genético. Sin embargo, como el conocimiento clínico/científico y la aprobación de terapias crece exponencialmente, y en el caso de que se incluyan posteriormente

al análisis inicial nuevas enfermedades con tratamiento, los datos genéticos de su bebé serán reanalizados durante el periodo del estudio, dos años,

- Resultado positivo: en el caso de que se detecte alguna alteración en el análisis bioquímico y/o genético, el personal sanitario incluido en este estudio llamará por teléfono a los padres y les dará cita para acudir a una consulta de seguimiento donde se informará a la familia sobre todos los aspectos de la enfermedad y prescribirá el tratamiento necesario para su bebé. Además, se ofrecerá cita para un adecuado asesoramiento genético.

Para complementar el cribado, se realizará un análisis metabolómico a partir de la misma muestra de sangre en papel, con el objetivo de poder identificar nuevos marcadores de las enfermedades cribadas.

- ¿Dónde se almacenarán las muestras y datos de su bebé?

Las muestras de sangre en papel seca se almacenarán en la Unidad de Metabolopatías, según práctica clínica habitual. Los datos genéticos obtenidos de la secuenciación del ADN de su bebé serán almacenados en el Centro de Supercomputación de Galicia (CESGA), donde se garantiza la seudonimización de sus datos personales.

Su participación tendrá una duración total estimada de 2 años.

El investigador puede decidir finalizar el estudio antes de lo previsto o interrumpir su participación. En todo caso se le informará de los motivos de su retirada.

¿Qué molestias o inconvenientes tiene?

Su participación no implica molestias adicionales a las de la práctica asistencial habitual para la toma de muestra del cribado bioquímico realizado de rutina a los recién nacidos en Galicia. En el caso de que haya un resultado positivo en el estudio genético, usted y su hijo tendrían que acudir al hospital en una visita de seguimiento adicional a la práctica clínica asistencial, para valoración clínica y asesoramiento familiar. Esta visita tendría la duración aproximada de 1 hora.

¿Obtendré algún beneficio por participar?

No se espera que ni su hijo ni ustedes obtengan beneficio directo por participar en el estudio. Se realizará un protocolo de cribado de más de 300 enfermedades genéticas a su hijo/a, basado en la secuenciación de exoma completo donde se podría encontrar la causa genética de una posible patología. Además, se espera que el conocimiento adquirido durante este proyecto piloto permita mejorar los programas de cribado neonatal, aumentando la detección muy temprana de enfermedades de baja prevalencia, y de debut infantil con tratamiento o intervención precoz disponible.

La investigación pretende descubrir aspectos desconocidos o poco claros sobre la ampliación de los programas de cribado neonatal que incluyen enfermedades de inicio en edad pediátrica con tratamiento disponible Esta información podrá ser de utilidad en un futuro para otras personas.

¿Recibiré la información que se obtenga del estudio?

Si Ud. Lo desea, se le facilitará un resumen de los resultados del estudio.

También podrá recibir los resultados de las pruebas que se realicen con sus muestras si así lo solicita dirigiéndose al investigador. Estos resultados pueden no tener aplicación clínica ni una interpretación clara, por lo que, si quiere disponer de ellos, deberían ser comentados con el médico del estudio.

Algunos resultados de pruebas genéticas podrían descubrir ciertas condiciones que afecten en el futuro a su salud. Estas condiciones podrían ser compartidas por sus familiares. Llegado el caso, sería conveniente que Ud. Les transmita esta información.

¿Se publicarán los resultados de este estudio?

Los resultados de este estudio serán remitidos a publicaciones científicas para su difusión, pero no se transmitirá ningún dato que pueda llevar a la identificación de los participantes.

Información referente a datos/muestras:

La obtención, tratamiento, conservación, comunicación y cesión de sus datos se hará conforme a lo dispuesto en el Reglamento General de Protección de Datos (Reglamento UE 2016-679 del Parlamento europeo y del Consejo, de 27 de abril de 2016) y la normativa española sobre protección de datos de carácter personal vigente.

La institución en la que se desarrolla esta investigación es la responsable del tratamiento de sus datos pudiendo contactar con el Delegado/a de Protección de Datos a través de los siguientes medios: correo electrónico: delegado.proteccion.datos@sergas.es

Los datos/muestras necesarios para llevar a cabo este estudio serán recogidos y conservados de modo:

• **Seudonimizados** (Codificados), la seudonimización es el tratamiento de datos personales de manera tal que no pueden atribuirse a un/a interesado/a sin que se use información adicional. En este estudio solamente el equipo investigador conocerá el código que permita saber su identidad.

La normativa que regula el tratamiento de datos de personas, le otorga el derecho a acceder a sus datos, oponerse, corregirlos, cancelarlos, limitar su tratamiento, restringir o solicitar la supresión de los mismos. También puede solicitar una copia de éstos o que ésta sea remitida a un tercero (derecho de portabilidad).

Para ejercer estos derechos puede Ud. Dirigirse al Delegado/a de Protección de Datos del centro a través de los medios de contacto antes indicados o al investigador/a principal de este estudio en el correo electrónico: maria.luz.couce.pico@sergas.es y/o tfno 981 950 162.

Así mismo, Ud. Tiene derecho a interponer una reclamación ante la Agencia Española de Protección de Datos, cuando considere que alguno de sus derechos no haya sido respetado.

Únicamente el equipo investigador y las autoridades sanitarias, que tienen el deber de guardar la confidencialidad, tendrán acceso a todos los datos recogidos en el estudio. Se podrá transmitir a terceros información que no pueda ser identificada. En el caso de que alguna información se transmita a otros países, se realizará con un nivel de protección de datos equivalente, como mínimo, al establecido por la normativa española y europea.

Al terminar este estudio, y conforme a la normativa, los datos generados serán

- **Seudonimizados (Codificados),** conservados en Centro de Supercomputación de Galicia (Cesga) en servidor protegido con acceso limitado a la IP del proyecto (María Luz Couce Pico). Los datos podrán ser utilizados para otros estudios en la misma línea de investigación previo informe favorable de un Comité de Ética de la Investigación. Los datos de identificación se mantendrán bajo la tutela del IP en un entorno SERGAS.

Usted podrá solicitar la destrución o anonimización de sus datos y los de su hijo/a en cualquier momento, dirigiéndose al investigador principal. Si bien, esto no será de aplicación a los datos resultantes de los análisis que ya hayan sido realizados

¿Existen intereses económicos en este estudio?

Esta investigación es promovida por Mª Luz Couce Pico con fondos aportados por el Instituto de Salud Carlos III (PI23/00276 y PIT23 CIBERER).

El investigador no recibirá retribución específica por la dedicación al estudio.

¿Cómo contactar con el equipo investigador de este estudio?

Ud. puede contactar con María Luz Couce Pico, IP del estudio, en el teléfono 981 950 162 y/o correo electrónico maría.luz.couce.pico@sergas.es y con su médico reclutador:

Nombre Médico reclutador: ________________________________

Teléfono: ________________________________

Correo electrónico: ________________________________

Muchas gracias por su colaboración.

Deberán firmarse tres modelos, uno será entregado al participante, otro será conservado en el centro en el que se obtuvo la muestra, y el tercero será conservado por el responsable del estudio de investigación

DOCUMENTO DE CONSENTIMIENTO PARA LOS PROGENITORES DE PARTICIPACIÓN EN UN ESTUDIO DE INVESTIGACIÓN

TÍTULO: ___________ESTUDIO PILOTO DE CRIBADO GENÉTICO NEONATAL: NBGENS__________

Yo, ____

- Leí la hoja de información al participante del estudio arriba mencionado que se me entregó, pude conversar con: ___________________________________ y hacer todas las preguntas sobre el estudio necesarias.
- Comprendo que la participación mía y de mi bebé es voluntaria, y que puedo retirarme del estudio cuando quiera, sin tener que dar explicaciones y sin que esto repercuta en mis cuidados médicos.
- Accedo a que se utilicen mis datos clínicos y los de mi bebé y sus muestras en las condiciones detalladas en la hoja de información al participante.
- Presto libremente mi conformidad para participar en este estudio.

Una vez terminado el estudio, LOS DATOS recogidas acepto que sean:

☐ Eliminados.

☐ Conservados seudonimizados en en el servidor habilitado para estos fines (CESGA)

Fdo.: Progenitor/a

(se firma con la connivencia del otro progenitor)

Nombre y apellidos: ____________________

Fecha:

Fdo.: El/la investigador/a que solicita el consentimiento

Nombre y apellidos: ____________________

Fecha:

Deberán firmarse tres modelos, uno será entregado al participante, otro será conservado en el centro en el que se obtuvo la muestra, y el tercero será conservado por el responsable del estudio de investigación

English translation

The following document is an English translation of the **original Spanish-language participant information sheet** and informed consent form approved by the Santiago-Lugo Research Ethics Committee of Galicia and used in the NBGenS study.

This translation is provided solely to facilitate editorial review and reader understanding. It was not used to obtain informed consent from participants and does not replace the original ethics-approved Spanish-language document.

INFORMATION SHEET FOR PARENTS/LEGAL GUARDIANS OF MINOR PARTICIPANTS

STUDY TITLE: Pilot Study of Newborn Genetic Screening: NBGenS

PRINCIPAL INVESTIGATOR María Luz Couce Pico
CENTRE: University Clinical Hospital of Santiago de Compostela

The purpose of this document is to provide you with information about a **research study** in which you and your child are being invited to participate. This study has been approved by the Santiago/Lugo Research Ethics Committee.

Before deciding whether to participate, you should receive personalized information from the investigator, **read this document carefully**, and ask any questions you may have in order to fully understand the details of the study. You may take this document with you, discuss it with other people, and take as much time as you need to decide whether or not to participate.

Participation in this study is entirely voluntary. You may choose not to participate or, if you initially agree, you may change your mind and withdraw your consent at any time without having to provide a reason. We assure you that this decision will not affect your relationship with the healthcare professionals responsible for your care or the healthcare services to which you and your child are entitled.

What is the purpose of the study?

Newborn screening is a public health programme designed to detect certain treatable congenital disorders in newborns—currently between 7 and 34 conditions in Spain—so that they can be identified early, before serious symptoms develop.

The purpose of this study is to implement an expanded newborn screening programme combining biochemical and genetic analysis in order to detect more than 300 rare, childhood-onset disorders for which early treatment or intervention is available.

The study also includes metabolomic analysis using the same sample. This may help identify new biochemical markers associated with the conditions included in the screening programme.

Why are my child and I being invited to participate?

You and your child are being invited to take part because your child meets the eligibility criteria for newborn genetic screening and is receiving care in the Obstetrics Department of the University Hospital Complex of Santiago de Compostela.

What does participation involve?

Your baby's participation in this study will involve:

- ▫ collection of clinical information at birth;
- ▫ metabolomic and genetic analyses performed using the dried blood spot sample, following DNA extraction where required.

The parents' participation in this study will involve:

- ▫ collection of clinical information and family medical history;
- ▫ completion of two questionnaires: one assessing knowledge of genetics and another assessing anxiety, on two occasions:
    1. i. At the beginning of the study, after providing consent for your own participation and that of your baby.
    2. ii. After the final report containing the screening results has been issued.

- When and how will my baby's samples be collected?

Blood samples will be collected as part of routine clinical care when your baby is between 24 and 48 hours old. The sample will be collected on filter paper at the same time as the sample used for conventional newborn screening, so no additional blood draw will normally be required.

A healthcare professional will collect the sample by performing a heel prick and placing drops of blood onto an approved absorbent filter-paper card. In accordance with routine clinical procedures, the sample will then be sent to the Metabolic Diseases Unit at the University Clinical Hospital of Santiago de Compostela.

The dried blood spot sample collected on the filter paper will be used for:

i. biochemical analysis as part of conventional newborn screening, which is included in routine healthcare;
ii. DNA extraction for genetic analysis; and
iii. untargeted metabolomic analysis.

In some cases, an additional sample may be required if the quality or quantity of the DNA extracted from the dried blood spot is insufficient to ensure reliable interpretation of the results.

- What will be analysed?

Both the biochemical and genetic analyses are intended to detect **rare**, childhood-onset disorders for which **treatments or early interventions are available** and may improve your baby's prognosis and development.

The biochemical analysis performed as part of conventional newborn screening allows the detection of 34 metabolic disorders

(https://www.sergas.es/Saude-publica/Patoloxias-incluidas-programa-galego-xeneticas).

For the genetic analysis, your baby's whole exome will be sequenced using the extracted DNA. This analysis will assess two groups of previously selected genes:

1. PedAC group, which includes genes associated with paediatric disorders that meet specific selection criteria. These genes are linked to well-established and well-characterised conditions for which treatment or early intervention is available and may improve the prognosis of affected children. This analysis is intended both to confirm biochemical screening results and to expand detection to approximately 300 conditions, including cardiac, endocrine, haematological, immunological, neurological, neuromuscular, metabolic, and hearing disorders.
2. Pharmacogenetics group, which includes genes associated with individual responses to medicines. This analysis aims to identify genetic variants that may affect how your child responds to certain medicines. This information may help healthcare professionals select medicines or doses that are safer and more appropriate for your child, potentially improving treatment effectiveness and reducing the risk of adverse reactions.

- How will the results be communicated?

The results of conventional biochemical newborn screening will be communicated in accordance with routine clinical practice, usually within one week. The family will receive the genetic study results by post at the address provided, approximately four weeks after sample collection. If the results have not been received within this period, you may report this to the Metabolic Diseases Laboratory by calling 981 950 100, Monday to Friday, from 8:30 a.m. to 2:30 p.m. Any other questions regarding the pilot study should be discussed with your child's paediatrician.

The possible outcomes of the genetic report generated as part of this study are:

- □ Negative result + pharmacogenetic findings
- □ Positive result + pharmacogenetic findings

The follow-up plan according to the report outcome is described below:

- □ Negative result: No abnormalities are detected in either the biochemical or genetic analysis. However, as clinical and scientific knowledge and the availability of approved treatments continue to expand, your baby's genetic data may be reanalysed during the two-year study period if new treatable conditions are added after the initial analysis.
- □ Positive result: If an abnormality is detected in the biochemical and/or genetic analysis, a member of the healthcare team involved in the study will contact the parents by telephone and arrange a follow-up appointment. During this consultation, the family will be informed about the condition, its implications, and the treatment or clinical management required for the baby. An appointment for appropriate genetic counselling will also be offered.

To complement the screening process, untargeted metabolomic analysis will also be performed using the same dried blood spot sample, with the aim of identifying new biomarkers associated with the conditions included in the screening programme.

- Where will my baby's samples and data be stored?

The dried blood spot samples will be stored at the Metabolic Diseases Unit in accordance with routine clinical practice. The genetic data obtained from sequencing your baby's DNA will be stored at the Galicia Supercomputing Centre (CESGA), where appropriate measures will be applied to ensure the pseudonymisation of personal data.

Your participation in the study is expected to last approximately two years.

The investigator may decide to end the study earlier than planned or to discontinue your participation. In either case, you will be informed of the reasons for this decision.

What discomforts or inconveniences are involved?

Participation in this study does not involve any additional discomfort beyond that associated with the routine blood sample collected for conventional newborn screening in Galicia.

If the genetic study produces a positive result, you and your child will be asked to attend an additional follow-up appointment at the hospital for clinical assessment and family counselling. This visit is expected to last approximately one hour.

Will my child or I receive any benefit from taking part?

No direct benefit to you or your child can be guaranteed as a result of participating in this study. Your child will undergo screening for more than 300 genetic disorders based on whole-exome sequencing, which may identify a genetic cause associated with a previously unrecognised condition.

In addition, the knowledge gained from this pilot project is expected to contribute to the improvement of newborn screening programmes by enabling the earlier detection of rare, childhood-onset disorders for which early treatment or intervention is available.

The research aims to address areas of uncertainty regarding the expansion of newborn screening programmes to include treatable disorders with onset during childhood. The knowledge generated may be useful in the future for other children and families.

Will I receive information obtained from the study?

At your request, you will be provided with a summary of the overall study results.

You may also request the results of tests performed using your child's samples by contacting the study investigator. Some of these results may have no established clinical relevance or may be difficult to interpret. For this reason, any individual results provided should be discussed with a study physician.

Some genetic findings may identify conditions that could affect your child's future health and may also be relevant to other family members. In such cases, it may be advisable to share this information with them, with support from the study team and appropriate genetic counselling.

Will the results of this study be published?

The results of this study may be submitted for publication in scientific journals and presented for scientific dissemination. No information that could identify individual participants will be disclosed.

Information regarding data and simples

The collection, processing, storage, communication, and transfer of your data will be carried out in accordance with the General Data Protection Regulation (Regulation (EU) 2016/679 of the European Parliament and of the Council of 27 April 2016) and the applicable Spanish legislation on the protection of personal data.

The institution where this research is being conducted is the data controller. You may contact the Data Protection Officer by email at: delegado.proteccion.datos@sergas.es

The data and samples required for this study will be collected and stored in pseudonymised (coded) form.

- **Pseudonymisation** means that personal data are processed in such a way that they cannot be attributed to a specific individual without the use of additional information. In this study, only authorised members of the research team will have access to the code linking the study data to your identity.

Data-protection legislation grants you the right to access your data, object to their processing, request correction or erasure, and request that their processing be limited or restricted. You may also request a copy of your data or ask for them to be transferred to a third party, where applicable (right to data portability).

To exercise these rights, you may contact the Data Protection Officer using the contact details provided above or the Principal Investigator of this study at: maria.luz.couce.pico@sergas.es and/or Telephone: 981 950 162.

You also have the right to lodge a complaint with the Spanish Data Protection Agency if you believe that any of your rights have not been respected.

Only the research team and the competent health authorities, all of whom are subject to a duty of confidentiality, will have access to the complete set of data collected during the study. Information that does not allow participants to be identified may be shared with third parties. If any information is transferred to other countries, it will be protected to a standard at least equivalent to that required under Spanish and European data-protection legislation.

At the end of the study, and in accordance with applicable regulations, the data generated will remain

- Pseudonymised (coded) and will be stored at the Galicia Supercomputing Centre (CESGA) on a secure server with access restricted to the project's authorised personnel under the responsibility of the Principal Investigator, María Luz Couce Pico. The data may be used in future studies within the same line of research, subject to prior approval by a Research Ethics Committee. Identifying information will remain under the responsibility of the Principal Investigator within a secure SERGAS environment.

You may request the destruction or anonymisation of your data and your child's data at any time by contacting the Principal Investigator. However, this will not apply to data or results derived from analyses that have already been completed.

¿Are there any financial interests associated with this study?

This research is sponsored by María Luz Couce Pico and is funded by the Instituto de Salud Carlos III through grants PI23/00276 and PIT23 CIBERER.

The investigator will not receive any specific payment for their involvement in the study.

How can I contact the study research team?

You may contact María Luz Couce Pico, the Principal Investigator of the study, by telephone at 981 950 162 and/or by email at maria.luz.couce.pico@sergas.es. You may also contact the physician who recruited you into the study:

Recruiting physician's name: ________________________________

Telephone: ________________________________

Email address: ______________________________________

Thank you very much for your participation.

PARENTAL CONSENT FORM FOR PARTICIPATION IN A RESEARCH STUDY

TITLE: __________PILOT STUDY OF NEWBORN GENETIC SCREENING: NBGENS__________

I, ____

- ◻ have read the participant information sheet for the above-mentioned study, which was provided to me, and have had the opportunity to speak with:
  _______________________________ and ask all the questions I considered necessary about the study.
- ◻ understand that my participation and my baby's participation are voluntary and that I may withdraw from the study at any time, without having to provide a reason and without this affecting the healthcare to which my baby or I are entitled.
- ◻ agree that my clinical data, my baby's clinical data, and the relevant samples may be used under the conditions described in the participant information sheet.
- ◻ freely consent to my participation and my baby's participation in this study.

Once the study has been completed, I agree that the data collected may be:

- ☐ Deleted.
- ☐ Retained in pseudonymised form on the secure server established for this purpose at the Galicia Supercomputing Centre (CESGA).

Signed by: Parent/Legal Guardian<br>(signed with the agreement<br>of the other parent/legal guardian)	Signed by: Investigator<br>obtaining consent
Full name: ___________________________	Full name: ___________________________
Date:	Date:

Deberán firmarse tres modelos, uno será entregado al participante, otro será conservado en el centro en el que se obtuvo la muestra, y el tercero será conservado por el responsable del estudio de investigación
