# Supplementary material for: The NBGENS project protocol: Pilot study for genetic newborn screening in Galicia
Source: PLoS One. 2026 Oct 8;21(10):e0359270. doi: 10.1371/journal.pone.0359270 (PMC13649029; doi:10.1371/journal.pone.0359270)
Supplement: Supplementary file 2 [file pone.0359270.s002.pdf]

¿Qué es el cribado neonatal?

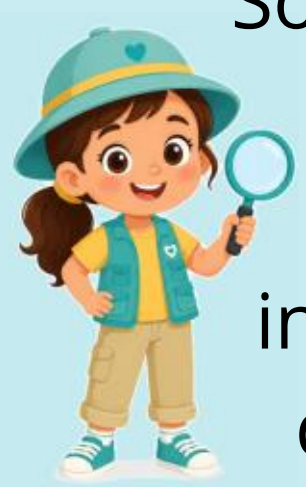

Son **pruebas simples** a recién nacidos para **detectar enfermedades** genéticas, incluso **antes** de la aparición de los **primeros síntomas**

Esto facilita que se puedan **iniciar tratamientos** y otras intervenciones **lo antes posible**

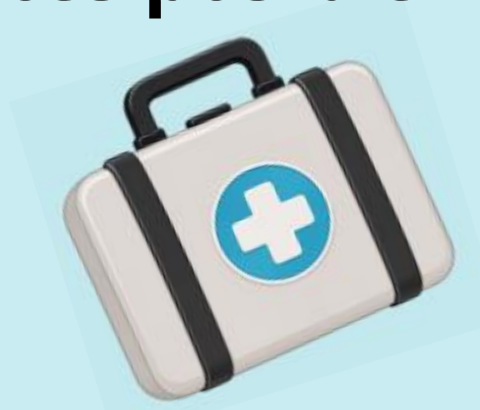

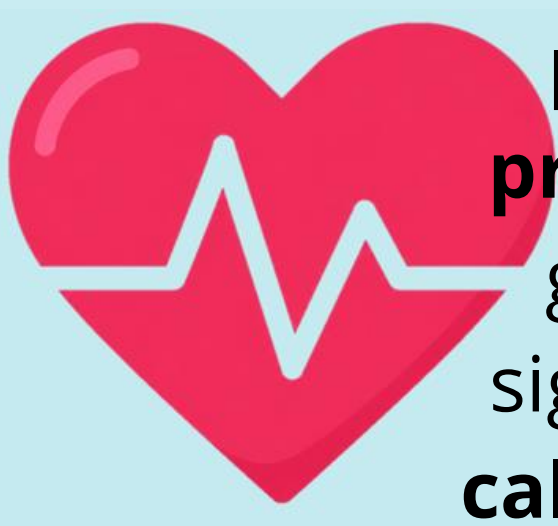

Si se **detectan a tiempo** podremos **evitar problemas** de salud graves y **mejorar** significativamente la **calidad de vida** de su hijo

NBGENS STUDY

En qué consiste nuestro estudio y cómo puede participar

1 Firmar consentimiento informado

2 Completar encuesta conocimiento genético

3 Se toma pequeña muestra de sangre del talón del bebé

4 Se extrae el ADN

5 Se analiza en un laboratorio especializado

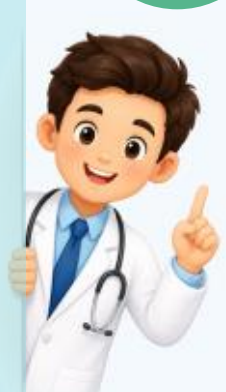

6 En pocas semanas se obtienen los **resultados**

CONFIDENCIALIDAD Y SEGURIDAD

Todos los datos de su hijo seguirán una estricta trazabilidad, serán anonimizados y se guardaran bajo alta seguridad

CONTACTO

Si tienes alguna duda o quieres contactar con nosotros:

Nbgens.chus@gmail.com

http:// udytemc.org/

INFORMACIÓN ADICIONAL

CÓDIGO QR DE WEB SOBRE NUESTRO NBGenS

¡Protege la salud de tu bebé desde el principio!

Resultados NORMALES

Se enviarán a casa

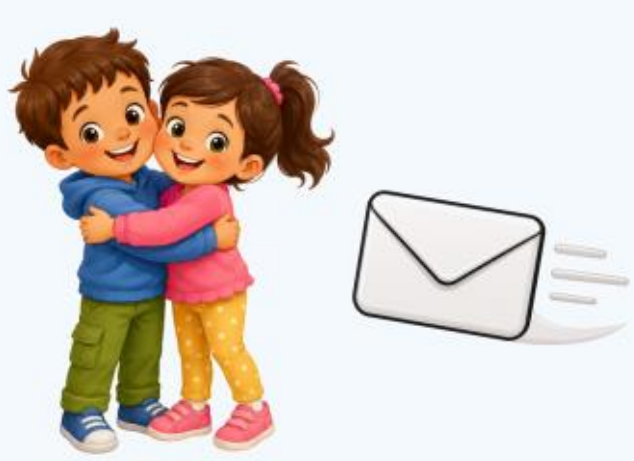

Resultados ANORMALES

ANORMAL NO SIGNIFICA SIEMPRE ENFERMO

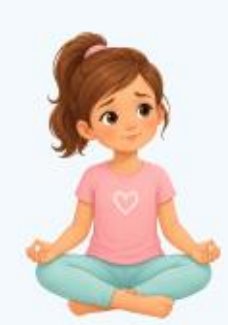

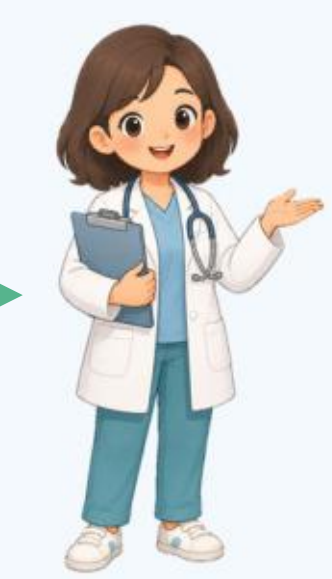

Serás citado por la unidad de **UDyTEMC**

What is Neonatal Genetic Screening?

These are **tests** conducted on newborns to **detect genetic disorders**, even before the **first symptoms** appear

This allows **treatments** and other interventions to be **initiated** as **early as possible**

If **detected early**, we can **prevent** serious health **issues** and significantly **improve** your child's **quality of life**

NBGENS STUDY

What does our study consist of, and how can you participate?

1. Sign the informed consent form
2. Complete the genetic knowledge survey
3. A small blood sample will be taken from the baby's heel
4. The DNA will be extracted
5. It will be analyzed in a specialized laboratory
6. **Results** will be available in a few weeks

NORMAL Results → Results will be sent to your home

ABNORMAL Results → You will be contacted by the **UDyTEMC** unit

ABNORMAL DOES NOT ALWAYS MEAN DISEASE

CONFIDENTIALITY AND SECURITY

All your child's data will follow strict traceability, will be anonymized, and stored under high security

CONTACT

If you have any questions or wish to contact us:

Nbgens.chus@gmail.com

http:// udytemc.org/

ADDITIONAL INFORMATION

WEB QR CODE ABOUT OUR NBGenS

Protect your baby's health from the start!

ما هو الفحص الجيني لحديثي الولادة ؟

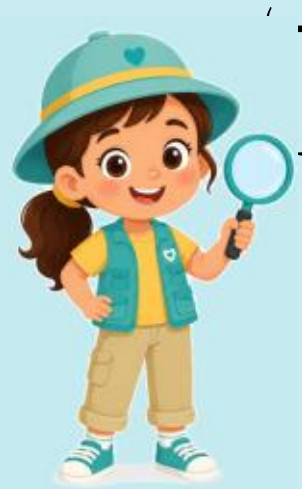

هي فحوصات تجري علي الاطفال للكشف عن الاضطرابات الوراثية، حتي قبل ظهو الأعراض الأولي

وهذا يسمح ببدء العلاج والتدخلات الأخرى في أقرب وقت ممكن.

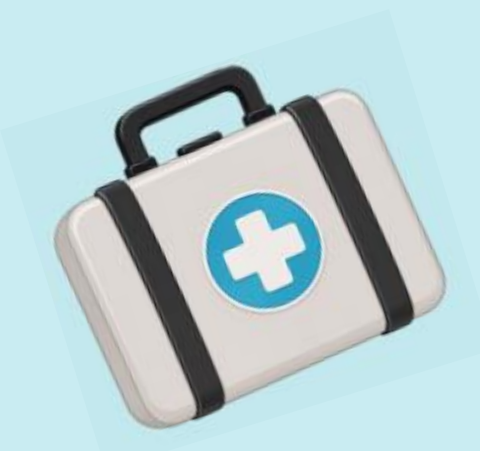

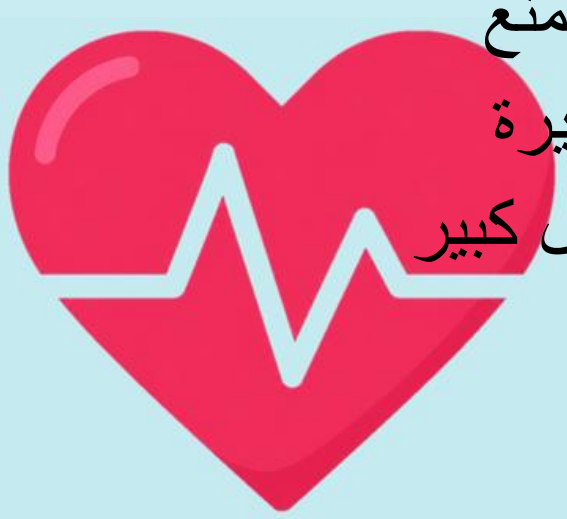

إذا تم اكتشافه مبكرًا، يمكننا منع حدوث مشكلات صحية خطيرة وتحسين نوعية حياة طفلك بشكل كبير

NBGENS STUDY

مما تتكون دراستنا وكيف يمكنك المشاركة؟

1 ١- التوقيع على نموذج الموافقة المستنيرة

2 ٢- استكمال استطلاع المعرفة الوراثية

3 ٣- سيتم أخذ عينة دم صغيرة من كعب الطفل

4 ٤- سيتم استخراج الحمض النووي

5 ٥- سيتم تحليلها في مختبر متخصص

6 ٦- ستكون النتائج متاحة خلال أسابيع قليلة

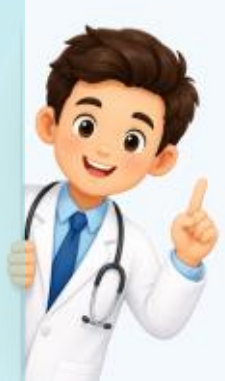

السرية والأمن

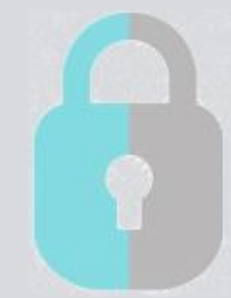

سيتم تتبعجميع بيانات طفلك بإمكانية التتبع الصارمة، وسيتم إخفاء هويتها وحفظها تحت إجراءات أمنية مشددة

اتصال

إذا كان لديك أي أسئلة أو ترغب في الاتصال بنا:

Nbgens.chus@gmail.com

http:// udytemc.org/

احمي صحة طفلك منذ البداية!

نتائج طبيعية.

نتائج غير طبيعية

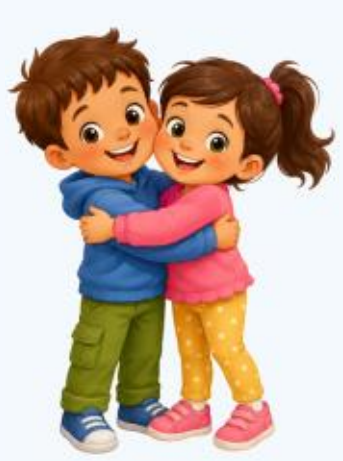

سيتم إرسال النتائج إلى منزلك

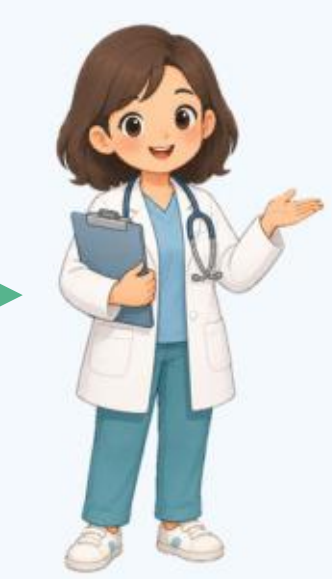

سيتم الاتصال UDyTEMC بك من قبل وحدة

النتائج غير الطبيعية لا تعني المرض في آغلب الآحيان

NBGENS STUDY

Pilot study of NewBorn genetic screening: The NBGenS

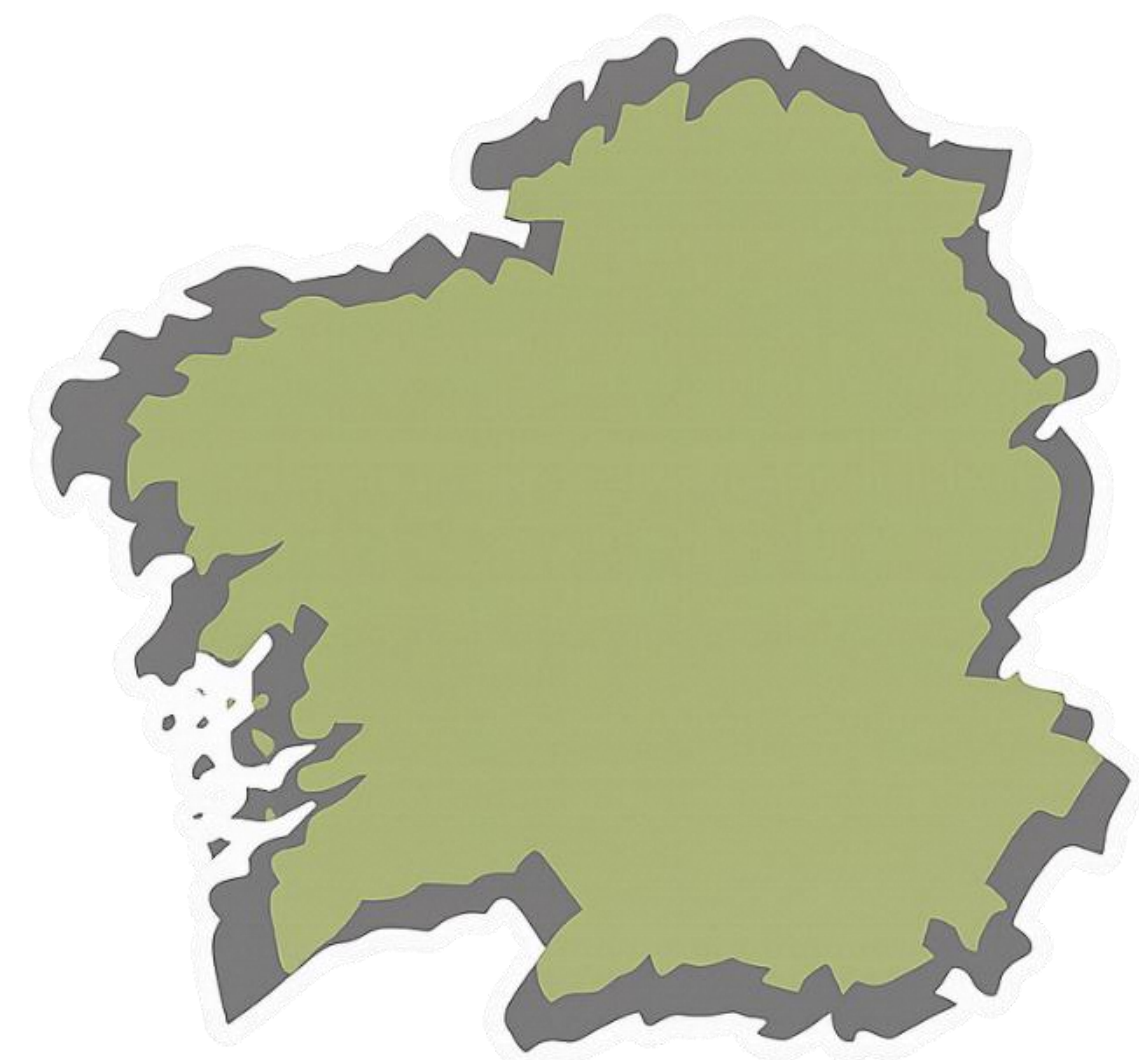

S2 Fig. The illustrative components of this figure were generated using OpenAI ChatGPT image generation (OpenAI, San Francisco, CA, USA). The AI-generated image was subsequently reviewed and edited by the authors, including the addition of labels and other graphical elements. Generative AI was used solely for the creation of the illustrative components and was not used to generate, modify, or interpret scientific data.
