# Supplementary material for: The NBGENS project protocol: Pilot study for genetic newborn screening in Galicia
Source: PLoS One. 2026 Oct 8;21(10):e0359270. doi: 10.1371/journal.pone.0359270 (PMC13649029; doi:10.1371/journal.pone.0359270)

Supplementary Figure. NBGenS reporting, reanalysis, recontact, and genomic data-management algorithm

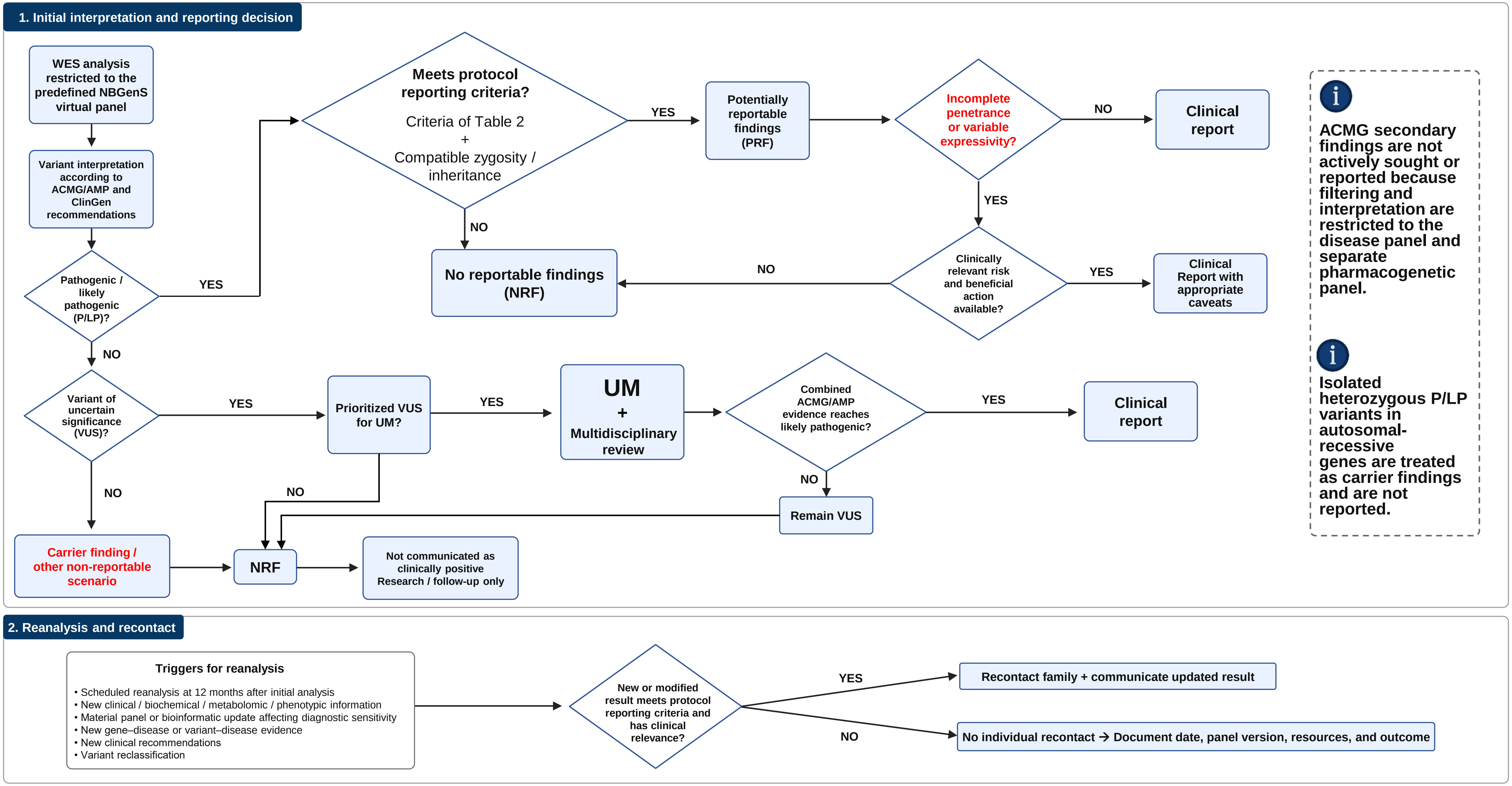

Supplementary Figure. NBGenS reporting, reanalysis, recontact, and genomic data-management algorithm

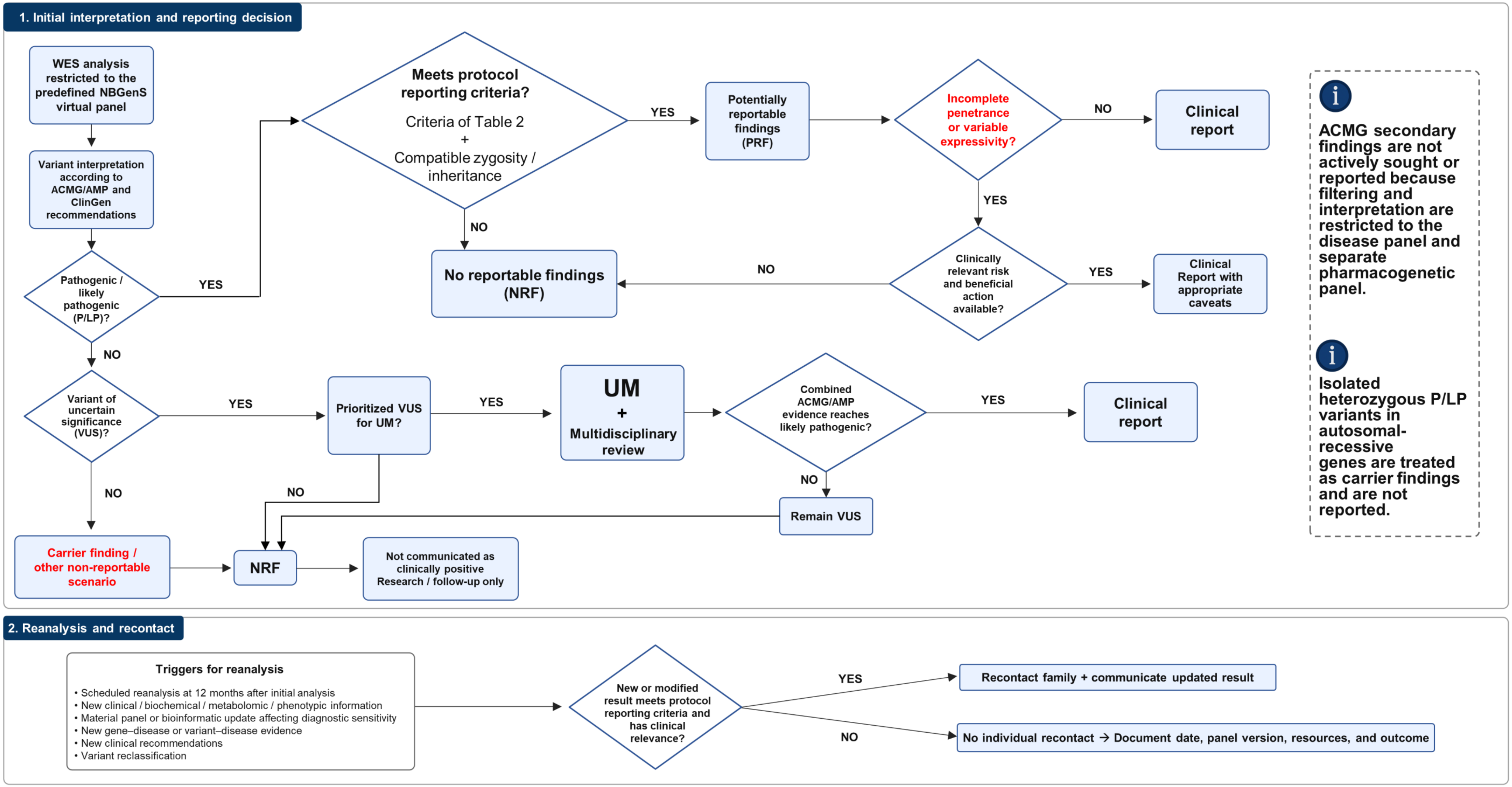

Supplement: Supplementary file 3 [file pone.0359270.s004.pdf]
