# Supplementary material for: The NBGENS project protocol: Pilot study for genetic newborn screening in Galicia
Source: PLoS One. 2026 Oct 8;21(10):e0359270. doi: 10.1371/journal.pone.0359270 (PMC13649029; doi:10.1371/journal.pone.0359270)
Supplement: Supplementary file 4 [file pone.0359270.s005.pdf]

**GENERAL SCHEME**

1. Paste the VarDB coverage data into the Word document [Word Coberturas_Informe].
2. Run the macro [Acondicionamiento Coberturas] in the Word document [Word Coberturas_Informe].
3. Complete the participants' information in the Excel file [Excel participantes_Informe].
4. Run the macro [Extracción coberturas de excel] in the Excel file [Excel participantes_Informe].
5. Run the macro [Crear informe no variantes] in the Word document [Modelo de informe no variante].

## MACRO [Acondicionamiento Coberturas]

```
Sub AcondicionamientoCoberturas()

  Dim doc As Document

  Set doc = ActiveDocument


  Dim muestraPositions As Collection

  Set muestraPositions = New Collection


  Dim rngBuscar As Range

  Set rngBuscar = doc.Content


  ' Recopilar posiciones de todas las ocurrencias "Muestra:"

  With rngBuscar.Find

    .Text = "Muestra:"

    .Forward = True

    .Wrap = wdFindStop

    .MatchCase = False

    .Execute

  End With


  Do While rngBuscar.Find.Found

    muestraPositions.Add rngBuscar.Start

    rngBuscar.Start = rngBuscar.Start + 1 ' Evitar bucle infinito

    rngBuscar.End = doc.Content.End

    rngBuscar.Find.Execute

  Loop


  Dim i As Long

  Dim startPos As Long, endPos As Long
```

```
    Dim casoRng As Range

    For i = 1 To muestraPositions.Count
        startPos = muestraPositions(i)
        If i < muestraPositions.Count Then
            endPos = muestraPositions(i + 1) - 1
        Else
            endPos = doc.Content.End
        End If

        ' Si el rango no es válido (start mayor que end), salir
        If startPos >= endPos Then Exit For

        Set casoRng = doc.Range(Start:=startPos, End:=endPos)

        ' Procesar en orden cada estructura
        Call SustituirHastaPunto(casoRng, "Mitocondrial:", ".")
        Call SustituirHastaPunto(casoRng, "Patología:", ".")
        Call SustituirHastaPunto(casoRng, "Informe:", ".")
        Call SustituirHastaPunto(casoRng, "Búsqueda de variantes mediante secuenciación NGS", ":")
        Call SustituirHastaPunto(casoRng, "Genes analizados en el panel virtual:", ".")
        Call SustituirHastaPunto(casoRng, "La región codificante del conjunto de los genes del exoma", ".")
    Next i

    MsgBox "Acondicionamiento completado."
End Sub


Sub SustituirHastaPunto(rngPadre As Range, textoClave As String, separador As String)
    Dim posClave As Long
    Dim buscarRng As Range
    Dim posSeparador As Long
    Dim reemplazarRng As Range

    Set buscarRng = rngPadre.Duplicate

    With buscarRng.Find
        .Text = textoClave
```

```
        .Forward = True
        .Wrap = wdFindStop
        .MatchCase = False
        .Execute
    End With

    Do While buscarRng.Find.Found And buscarRng.Start < rngPadre.End
        posClave = buscarRng.Start

        Dim revRng As Range
        Set revRng = rngPadre.Document.Range(rngPadre.Start, posClave)

        Dim sepPos As Long
        sepPos = InStrRev(revRng.Text, separador)

        If sepPos > 0 Then
            Dim absSepPos As Long
            absSepPos = revRng.Start + sepPos - 1

            Set reemplazarRng = rngPadre.Document.Range(absSepPos + 1, posClave)
            reemplazarRng.Text = " "
        End If

        buscarRng.Start = posClave + Len(textoClave)
        buscarRng.End = rngPadre.End
        buscarRng.Find.Execute
    Loop
End Sub
```

# MACRO [Extracción coberturas de excel]

```
Sub ExtracciónCoberturasdeExcel()


    Dim docWord As Object

    Dim rutaWord As String

    Dim rangoTexto As Object

    Dim textoDespuesDeMuestra As String

    Dim foundRange As Object

    Dim appExcel As Object

    Dim wsExcel As Object

    Dim comparadorSheet As Object

    Dim posicionComa As Long

    Dim codigoXenomica As String

    Dim columna As Long ' Para controlar la columna donde se pega el texto

    Dim encontrado As Boolean

    Dim inicioTexto As Long, finTexto As Long

    Dim fragmentoCompleto As String

    Dim cobertura1 As String, cobertura2 As String, cobertura3 As String, cobertura4 As String

    Dim lastRow As Long

    Dim i As Long

    Dim j As Long


    ' Ruta del documento Word

    rutaWord = "Ruta del Word Coberturas_Informe"


    ' Abre el documento Word

    On Error Resume Next

    Set docWord = GetObject(rutaWord) ' Intenta obtener el documento si está abierto

    If docWord Is Nothing Then

        Set docWord = CreateObject("Word.Application").Documents.Open(rutaWord)

    End If

    On Error GoTo 0


    If docWord Is Nothing Then

        MsgBox "No se pudo abrir el archivo de Word. Verifica la ruta."

        Exit Sub

    End If
```

```
' Asegúrate de que la ventana de Word esté visible
docWord.Application.Visible = True

' Establece el rango para buscar en todo el documento
Set rangoTexto = docWord.Content
columna = 1 ' Comenzamos en la columna 1 (A)
encontrado = True ' Para controlar el bucle

' Obtener la hoja activa
Set appExcel = GetObject(, "Excel.Application")
Set wsExcel = appExcel.ActiveWorkbook.Sheets(1)
Set comparadorSheet = appExcel.ActiveWorkbook.Sheets("COMPARADOR (NO TOCAR)")

' Encontrar la última fila ocupada en la columna N
lastRow = wsExcel.Cells(wsExcel.Rows.Count, "N").End(xlUp).Row

' Buscar mientras se encuentre "Muestra:"
Do While encontrado
    ' Buscar la palabra "Muestra:"
    With rangoTexto.Find
        .text = "Muestra:"
        .Forward = True
        .Wrap = wdFindStop
        .MatchCase = False
        .Execute
    End With

    ' Si se encuentra "Muestra:"
    If rangoTexto.Find.Found Then
        ' Define el rango después de "Muestra:"
        Set foundRange = docWord.Range(rangoTexto.End, docWord.Content.End)

        ' Extraer los primeros 8 caracteres después de "Muestra:"
        textoDespuesDeMuestra = Left(foundRange.text, 8)

        ' Almacenar el texto en "COMPARADOR (NO TOCAR)"
        comparadorSheet.Cells(2, columna).Value = Trim(textoDespuesDeMuestra) ' Columna A o B
```

```
' Comparar el valor extraído con cada celda en la columna N
For i = 2 To lastRow
    codigoXenomica = wsExcel.Cells(i, "N").Value

    If Trim(textoDespuesDeMuestra) = Trim(codigoXenomica) Then
        ' Extraer fragmento completo para coberturas
        Set rng = docWord.Range(rangoTexto.End, docWord.Content.End)
        text = rng.text

        ' Buscar el inicio y el final del fragmento que nos interesa
        inicioTexto = InStr(text, "La región codificante de los genes incluidos en el panel virtual presenta")
        finTexto = InStr(text, "El cálculo de la cobertura de análisis")

        If inicioTexto > 0 And finTexto > 0 Then
            ' Extraer el fragmento completo
            fragmentoCompleto = Mid(text, inicioTexto, finTexto - inicioTexto)

            ' Extraer las coberturas según el criterio especificado
            ' Cobertura1 (hasta el primer 100%)
            cobertura1 = Mid(fragmentoCompleto, 1, InStr(fragmentoCompleto, "100%") + 3)

            ' Cobertura2 (entre el 100% y el primer 95%)
            Dim inicioK As Long
            inicioK = InStr(fragmentoCompleto, "100%") + 4
            cobertura2 = Mid(fragmentoCompleto, inicioK, InStr(inicioK, fragmentoCompleto, "95%") - inicioK + 3)

            ' Cobertura3 (entre el 95% y el primer 90%)
            Dim inicioL As Long
            inicioL = InStr(fragmentoCompleto, "95%") + 3
            cobertura3 = Mid(fragmentoCompleto, inicioL, InStr(inicioL, fragmentoCompleto, "90%") - inicioL + 3)

            ' Cobertura4 (desde el 90% hasta el final del fragmento)
            Dim inicioM As Long
            inicioM = InStr(fragmentoCompleto, "90%") + 3
            cobertura4 = Mid(fragmentoCompleto, inicioM)

            ' Colocar los valores extraídos en las columnas J, K, L, M de la fila correspondiente en "Hoja1"
            wsExcel.Cells(i, 10).Value = Trim(cobertura1) ' Columna J - Cobertura1
```

```
                wsExcel.Cells(i, 11).Value = Trim(cobertura2) ' Columna K - Cobertura2

                wsExcel.Cells(i, 12).Value = Trim(cobertura3) ' Columna L - Cobertura3

                wsExcel.Cells(i, 13).Value = Trim(cobertura4) ' Columna M - Cobertura4

            Else

                ' Si no se encuentra el fragmento adecuado, dejar las celdas en blanco

                wsExcel.Cells(i, 10).Value = "?"

                wsExcel.Cells(i, 11).Value = "?"

                wsExcel.Cells(i, 12).Value = "?"

                wsExcel.Cells(i, 13).Value = "?"

            End If


            ' Una vez que se encuentra una coincidencia, no es necesario seguir buscando

            Exit For

          End If

        Next i


        ' Preparar para la siguiente columna

        columna = columna + 1


        ' Actualizar rango de búsqueda para la siguiente iteración

        rangoTexto.Start = rangoTexto.End

    Else

        encontrado = False ' No se encontró más "Muestra:"

    End If

  Loop


  MsgBox "Extracción completada."


End Sub
```

# MACRO [Crear informe no variantes]

```
Sub CrearInformeNoVariantes()

    Dim appExcel As Object

    Dim wbExcel As Object

    Dim wsExcel As Object

    Dim nombre As String

    Dim referencia As String

    Dim sexo As String

    Dim nacimiento As String

    Dim direccion As String

    Dim cp As String

    Dim poblacion As String

    Dim provincia As String

    Dim cobertura1 As String

    Dim cobertura2 As String

    Dim cobertura3 As String

    Dim cobertura4 As String

    Dim fecha As String

    Dim fechaFija As String

    Dim docWordOriginal As Document

    Dim docWordModificado As Document

    Dim rutaExcel As String

    Dim rutaWordOriginal As String

    Dim rutaWordGuardado As String

    Dim rutaWordCopia As String

    Dim fso As Object

    Dim i As Integer

    Dim ultimaFila As Long

    Dim shape As shape


    ' Definir las rutas de los archivos

    rutaExcel = "Ruta del Excel Participantes_Informe"

    rutaWordOriginal = "Ruta del Word Modelo de informe no variante"


    ' Definir la ruta para la copia del documento original

    rutaWordCopia = "Ruta para generar la copia del documento original"


    ' Inicializa la aplicación de Excel
```

```
On Error Resume Next
Set appExcel = GetObject(, "Excel.Application")
If appExcel Is Nothing Then
    Set appExcel = CreateObject("Excel.Application")
End If
On Error GoTo 0

' Abre el archivo de Excel
On Error Resume Next
Set wbExcel = appExcel.Workbooks.Open(rutaExcel)
If wbExcel Is Nothing Then
    MsgBox "No se pudo abrir el archivo de Excel. Verifica la ruta."
    Exit Sub
End If
Set wsExcel = wbExcel.Sheets("Hoja1")
If wsExcel Is Nothing Then
    MsgBox "No se pudo acceder a la hoja de Excel. Verifica el nombre de la hoja."
    wbExcel.Close False
    Exit Sub
End If

' Obtener el valor fijo de la celda E2 para la fecha
fechaFija = wsExcel.Range("E2").Value

' Encontrar la última fila con datos
ultimaFila = wsExcel.Cells(wsExcel.Rows.Count, "A").End(xlUp).Row

' Crear un objeto FileSystemObject para copiar y eliminar archivos
Set fso = CreateObject("Scripting.FileSystemObject")

' Iterar sobre cada fila de datos
For i = 2 To ultimaFila
    ' Obtén los valores de la fila actual
    nombre = wsExcel.Cells(i, 1).Value
    referencia = wsExcel.Cells(i, 2).Value
    sexo = wsExcel.Cells(i, 3).Value
    nacimiento = wsExcel.Cells(i, 4).Value
    direccion = wsExcel.Cells(i, 6).Value
```

```
cp = wsExcel.Cells(i, 7).Value
poblacion = wsExcel.Cells(i, 8).Value
provincia = wsExcel.Cells(i, 9).Value
cobertura1 = wsExcel.Cells(i, 10).Value ' Columna J para Cobertura1
cobertura2 = wsExcel.Cells(i, 11).Value ' Columna K para Cobertura2
cobertura3 = wsExcel.Cells(i, 12).Value ' Columna L para Cobertura3
cobertura4 = wsExcel.Cells(i, 13).Value ' Columna M para Cobertura4

' Crear una copia del documento original
On Error Resume Next
fso.CopyFile rutaWordOriginal, rutaWordCopia, True
If Not fso.FileExists(rutaWordCopia) Then
    MsgBox "No se pudo crear la copia del archivo de Word. Verifica la ruta."
    Exit Sub
End If

' Abre la copia del documento de Word
On Error Resume Next
Set docWordModificado = Documents.Open(rutaWordCopia)
If docWordModificado Is Nothing Then
    MsgBox "No se pudo abrir el documento de Word copiado."
    Exit Sub
End If

' Reemplaza los términos en el documento modificado
With docWordModificado.Content.Find
    .Text = "<<Nombre>>"
    .Replacement.Text = nombre
    .Forward = True
    .Wrap = 1 ' wdFindContinue
    .Format = False
    .Execute Replace:=2 ' wdReplaceAll

    .Text = "<<Referencia>>"
    .Replacement.Text = referencia
    .Execute Replace:=2

    .Text = "<<Sexo>>"
```

```
    .Replacement.Text = sexo
    .Execute Replace:=2

    .Text = "<<Nacimiento>>"
    .Replacement.Text = nacimiento
    .Execute Replace:=2

    .Text = "<<Direccion>>"
    .Replacement.Text = direccion
    .Execute Replace:=2

    .Text = "<<CP>>"
    .Replacement.Text = cp
    .Execute Replace:=2

    .Text = "<<Poblacion>>"
    .Replacement.Text = poblacion
    .Execute Replace:=2

    .Text = "<<Provincia>>"
    .Replacement.Text = provincia
    .Execute Replace:=2

    .Text = "<<Fecha>>"
    .Replacement.Text = fechaFija
    .Execute Replace:=2
End With

' Reemplazar dentro de todos los cuadros de texto
For Each shape In docWordModificado.Shapes
    If shape.Type = msoTextBox Then
        With shape.TextFrame.TextRange.Find
            .Text = "<<Nombre>>"
            .Replacement.Text = nombre
            .Forward = True
            .Wrap = 1 ' wdFindContinue
            .Format = False
            .Execute Replace:=2 ' wdReplaceAll
```

```
    .Text = "<<Direccion>>"
    .Replacement.Text = direccion
    .Execute Replace:=2

    .Text = "<<CP>>"
    .Replacement.Text = cp
    .Execute Replace:=2

    .Text = "<<Poblacion>>"
    .Replacement.Text = poblacion
    .Execute Replace:=2

    .Text = "<<Provincia>>"
    .Replacement.Text = provincia
    .Execute Replace:=2

    ' Reemplazo de los campos <<Cobertura1>> hasta <<Cobertura4>>
    .Text = "<<Cobertura1>>"
    .Replacement.Text = cobertura1
    .Execute Replace:=2

    .Text = "<<Cobertura2>>"
    .Replacement.Text = cobertura2
    .Execute Replace:=2

    .Text = "<<Cobertura3>>"
    .Replacement.Text = cobertura3
    .Execute Replace:=2

    .Text = "<<Cobertura4>>"
    .Replacement.Text = cobertura4
    .Execute Replace:=2

    .Text = "<<Fecha>>"
    .Replacement.Text = fechaFija
    .Execute Replace:=2
End With
```

```
            End If
        Next shape

        ' Define la ruta para guardar el documento modificado
        rutaWordGuardado = "Ruta para guardar el informe en word” & referencia & "_InformeNoVariantesV2_" & fechaFija & ".docx"
        rutaPDFGuardado = "Ruta para guardar el informe en pdf" & referencia & "_InformeNoVariantesV2_" & fechaFija & ".pdf"

        ' Guarda el documento modificado con el nuevo nombre
        docWordModificado.SaveAs2 rutaWordGuardado, wdFormatDocumentDefault
        docWordModificado.ExportAsFixedFormat OutputFileName:=rutaPDFGuardado, ExportFormat:=wdExportFormatPDF
        docWordModificado.Close SaveChanges:=False

        ' Elimina la copia temporal del documento original
        On Error Resume Next
        fso.DeleteFile rutaWordCopia
        On Error GoTo 0

        ' Libera el objeto del documento
        Set docWordModificado = Nothing
    Next i

    ' Cierra el archivo de Excel
    wbExcel.Close False
    Set wbExcel = Nothing
    Set wsExcel = Nothing
    Set appExcel = Nothing

    MsgBox "Informes creados y documentos guardados."
End Sub
```
