# Supplementary material for: The NBGENS project protocol: Pilot study for genetic newborn screening in Galicia
Source: PLoS One. 2026 Oct 8;21(10):e0359270. doi: 10.1371/journal.pone.0359270 (PMC13649029; doi:10.1371/journal.pone.0359270)
Supplement: Supplementary file 5 [file pone.0359270.s008.docx]

**Response to the Editor and Reviewer**

**Manuscript title:** “The NBGENS project protocol: pilot study for genetic Newborn screening in Galicia”
**Manuscript ID:** [PONE-D-26-17272] - [EMID:edc2e8f902847a27]

Dear Editor and Reviewer,

We sincerely thank the Editor and the Reviewer for their careful evaluation of our manuscript and for their constructive comments and suggestions. We have carefully considered all the points raised and have revised the manuscript accordingly.

Below, we provide a detailed point-by-point response. The Reviewer’s comments are reproduced in **bold**, followed by our responses. All changes introduced in the revised manuscript have been highlighted using [TRACK CHANGES]. Page and line numbers refer to the revised version of the manuscript.

**Response to the Editor**

**Editor’s comment 1**

**Please ensure that your manuscript meets PLOS ONE's style requirements, including those for file naming. The PLOS ONE style templates can be found at https://journals.plos.org/plosone/s/file?id=wjVg/PLOSOne_formatting_sample_main_body.pdf and https://journals.plos.org/plosone/s/file?id=ba62/PLOSOne_formatting_sample_title_authors_affiliations.pdf**

**Response:**
We thank the Editor for this guidance. We have carefully reviewed the PLOS ONE formatting and file-naming requirements and have revised the manuscript and all associated submission files accordingly.

In particular, we corrected the designation of the Supporting Information files. In the previous submission, all supplementary materials were incorrectly labelled as figures. Each file has now been assigned the appropriate designation—“Fig”, “File”, or “Table”—according to its content. These designations have been corrected consistently in: the names of the Supporting Information files; the citations to the Supporting Information within the main text; and the Supporting Information captions and list included in the manuscript.

These modifications can be found on page (lines): S1-11 (275); S3-17 (421;427); S4-19 (458); S6-23 (571); S7-25 (634); and throughout the revised Supporting Information files on page 39, lines 959-976.

Following the addition of the new supplementary figure requested by the Reviewer, the subsequent supplementary figures have been renumbered accordingly. All corresponding citations in the manuscript and Supporting Information have also been updated to ensure consistency.

**Editor’s comment 2**

**We note that the grant information you provided in the ‘Funding Information’ and ‘Financial Disclosure’ sections do not match. When you resubmit, please ensure that you provide the correct grant numbers for the awards you received for your study in the ‘Funding Information’ section.**

**Response:**
We thank the Editor for identifying this inconsistency. We have reviewed the funding information provided during the submission process and corrected the discrepancy between the “Funding Information” and “Financial Disclosure” sections.

The corresponding grant information and award numbers are now reported consistently and accurately in the online submission system.

**Editor’s comment 3**

**Thank you for stating the following financial disclosure: [Carlos III Health Institute (ISCIII by PI23/00276). CIBERER (Convocatoria Proyectos de Investigacion Traslacional 2023)]. Please state what role the funders took in the study. If the funders had no role, please state: "The funders had no role in study design, data collection and analysis, decision to publish, or preparation of the manuscript."**

**If this statement is not correct you must amend it as needed. Please include this amended Role of Funder statement in your cover letter; we will change the online submission form on your behalf.**

**Response:**
We appreciate the Editor’s request for clarification regarding the role of the funders. The funders had no involvement in the design or conduct of the study, the analysis of the data, the decision to publish, or the preparation of the manuscript.

Accordingly, we have included the following statement in the Financial Disclosure section: “The funders had no role in study design, data collection and analysis, decision to publish, or preparation of the manuscript.”

The same statement has also been included in the revised cover letter, as requested.

**Editor’s comment 4**

**Please remove any funding-related text from the manuscript. Please note that funding information should not appear in any section or other areas of your manuscript. We will publish the funding statement that is present in the Financial Disclosure section of the online submission form.**

**Response:**
We thank the Editor for this clarification. In accordance with the journal’s instructions, we have removed all funding-related information from the manuscript.

Specifically, we deleted:

- the “Funding” section previously included on page 1, line 24; and
- the funding-related reference to CIBERER previously included on page 9, lines 230–231.

Funding information is now provided exclusively through the Financial Disclosure section of the online submission system.

**Editor’s comment 5**

**Thank you for providing your underlying data as Supporting Information. We note that the data set contains text or data that is not in English. Please note that PLOS is an English-language publisher, so we require data sets to be provided in English as well. Please upload an English-language version of your data set.**

**This will also allow us to determine if your data follows PLOS standards per our Data Availability policy here:** [**https://journals.plos.org/plosone/s/data-availability**](https://journals.plos.org/plosone/s/data-availability)

**Response:**
We thank the Editor for highlighting this requirement. We have carefully reviewed all Supporting Information files and ensured that all relevant content is now available in English. Where the original material was required to remain in Spanish, a complete English translation has been provided alongside the original version.

The principal modifications include the following:

- An English translation of the informed consent form has been added to Supporting Information File S1, alongside the original Spanish-language version. This has also been clarified in the manuscript on page 11, lines 271-277: “Once the informed consent form has been signed, the newborn is considered fully enrolled in the study, and a copy of the signed document is provided to the family. The Spanish-language participant information sheet and informed consent form approved by the Research Ethics Committee, which will be used during recruitment, together with an English translation supplied solely for readers, are included in S1 File.”
- All non-English textual elements in Supporting Information File 6, including the relevant code annotations, labels, and explanatory content, have been translated into English.
- Supporting Information Table S7 has been fully translated into English and reorganized in accordance with the additional comments provided during review.

The revised Supporting Information files have been uploaded with the resubmission.

**Editor’s comment 6**

**We note that Figure S2 in your submission contains copyrighted images. All PLOS content is published under the Creative Commons Attribution License (CC BY 4.0), which means that the manuscript, images, and Supporting Information files will be freely available online, and any third party is permitted to access, download, copy, distribute, and use these materials in any way, even commercially, with proper attribution. For more information, see our copyright guidelines: http://journals.plos.org/plosone/s/licenses-and-copyright.**

**We require you to either (1) present written permission from the copyright holder to publish these figures specifically under the CC BY 4.0 license, or (2) remove the figures from your submission:**

**1. You may seek permission from the original copyright holder of Figure S2 to publish the content specifically under the CC BY 4.0 license. We recommend that you contact the original copyright holder with the Content Permission Form (http://journals.plos.org/plosone/s/file?id=7c09/content-permission-form.pdf) and the following text: “I request permission for the open-access journal PLOS ONE to publish XXX under the Creative Commons Attribution License (CCAL) CC BY 4.0 (http://creativecommons.org/licenses/by/4.0/). Please be aware that this license allows unrestricted use and distribution, even commercially, by third parties. Please reply and provide explicit written permission to publish XXX under a CC BY license and complete the attached form.” Please upload the completed Content Permission Form or other proof of granted permissions as an "Other" file with your submission. In the figure caption of the copyrighted figure, please include the following text: “Reprinted from [ref] under a CC BY license, with permission from [name of publisher], original copyright [original copyright year].”**

**2. If you are unable to obtain permission from the original copyright holder to publish these figures under the CC BY 4.0 license or if the copyright holder’s requirements are incompatible with the CC BY 4.0 license, please either i) remove the figure or ii) supply a replacement figure that complies with the CC BY 4.0 license. Please check copyright information on all replacement figures and update the figure caption with source information. If applicable, please specify in the figure caption text when a figure is similar but not identical to the original image and is therefore for illustrative purposes only.**

**Response:**
We thank the Editor for drawing our attention to the copyright requirements applicable to Supporting Information Figure S2.

In response to both of the requests received from the journal, we have selected the second option indicated in the original communication and have provided a fully revised replacement version of S2 Fig that is compatible with publication under the CC BY 4.0 license.

The following changes have been made:

- Removal of logos. All logos and brand-related graphic elements have been removed from the revised figure, including the NBGenS project logo, the institutional logos, and the YouTube logo previously embedded within a QR code. Where necessary, project name is now presented only as plain text.
- Removal of Canva graphic content. The original Canva-sourced map of Galicia and any other graphic elements originating from the Canva library have been removed.
- Reconstruction of the figure. The revised brochure was recreated by the authors in Microsoft PowerPoint. PowerPoint was used solely as a layout tool, using native text boxes, standard fonts, solid-colour backgrounds, circles, rectangles, lines, arrows, and other basic geometric elements. No Microsoft stock images, icons, clipart, templates, or other third-party visual resources were used.
- Source of the illustrative images. All remaining non-data-bearing illustrations were generated specifically for S2 Fig by the authors using ChatGPT (OpenAI), based on original prompts written by the authors. These illustrations were not obtained from Canva, image banks, clipart collections, or other third-party sources. All generated images were reviewed, selected, and edited by the authors.

Under OpenAI’s applicable Terms of Use, as between the user and OpenAI and to the extent permitted by applicable law, the user owns the generated Output, and OpenAI assigns to the user any rights, title, and interest it may have in that Output. The relevant terms are available at https://openai.com/policies/eu-terms-of-use/“Artificial intelligence disclosure” in pages 32-33, lines 800-802: The manuscript has been updated to clarify that ChatGPT was used not only for language editing but also to generate the non-data-bearing illustrative images used in S2 Fig. ChatGPT was not used to generate, alter, analyse, or interpret any study data or scientific results.

To the best of our knowledge, the revised S2 Fig contains no third-party visual content requiring copyright permission. The authors authorize the complete revised figure to be published and reused under the CC BY 4.0 license. Consequently, no Content Permission Form is applicable to the revised replacement figure.

**Additional editorial guidance**

**If the reviewer comments include a recommendation to cite specific previously published works, please review and evaluate these publications to determine whether they are relevant and should be cited. There is no requirement to cite these works unless the editor has indicated otherwise.**

**Response:**
We thank the Editor for this clarification. We have taken this guidance into account when addressing the Reviewer’s comments. Any publications recommended by the Reviewer have been independently assessed for their relevance to the scope, methodology, and scientific context of the present protocol. References have only been added when they were considered directly relevant and appropriate to support or clarify the revised manuscript.

**Response to Reviewer 1**

We sincerely thank the Reviewer for the careful and constructive assessment of our manuscript. The comments raised have allowed us to identify several aspects of the protocol that required further methodological and clinical clarification. We have revised the manuscript accordingly, as detailed below. Page and line numbers refer to the revised version of the manuscript.

**Major Comments**

**Major Comment 1**

**The authors state: "In cases where abnormalities associated with a VUS are detected, the variant would be reclassified as likely pathogenic and reported." This statement is clinically incorrect and inconsistent with ACMG/AMP variant classification guidelines (Richards et al., Genet Med 2015), which the authors simultaneously cite as their interpretive framework.**

**Under ACMG/AMP criteria, variant reclassification from VUS to likely pathogenic requires integrated assessment of multiple evidence streams: population frequency data, in silico predictions, segregation analysis, case-level evidence, and functional data. A single metabolomic signal constitutes functional evidence under the PS3 criterion but is insufficient in isolation to support reclassification. Applying this policy as written risks significant over-reporting with direct clinical consequences for asymptomatic newborns and their families, and could cause clinical harm through false-positive reporting in a screening context.**

**The authors must revise this section to explicitly state that UM findings represent one line of evidence within a structured ACMG/AMP classification framework, describe the multidisciplinary variant review process that will govern reclassification decisions, and specify which combination of ACMG criteria must be met before a VUS is upgraded to likely pathogenic.**

**Response:**
We thank the Reviewer for identifying this clinically important inconsistency. We agree that the original wording was overly categorical and could be interpreted as implying that an abnormal untargeted metabolomics result would automatically lead to VUS reclassification and clinical reporting, which was not the intended policy.

We have revised the manuscript to clarify that untargeted metabolomics provides an additional source of evidence within the structured ACMG/AMP classification framework but cannot, in isolation, determine variant reclassification or clinical communication. When a VUS is identified together with an untargeted metabolomic profile compatible with the disorder under evaluation, the applicability of the metabolomic finding as phenotypic or functional evidence—for example, PP4 or PS3, respectively—and the strength assigned will be determined according to the disease mechanism, the specificity of the metabolic profile, and the degree of analytical and clinical validation of the assay, in accordance with ACMG/AMP and ClinGen recommendations. Correlated evidence will not be counted more than once.

All potential reclassification decisions will be reviewed by a multidisciplinary committee integrating population, computational, segregation, phenotypic, functional, and case-level evidence. A VUS will be reclassified as likely pathogenic only when the combined independent evidence meets one of the ACMG/AMP combinations established for that category—one very strong and one moderate criterion; one strong and one or two moderate criteria; one strong and at least two supporting criteria; at least three moderate criteria; two moderate and at least two supporting criteria; or one moderate and at least four supporting criteria—and no relevant contradictory evidence is present. Clinical communication will additionally require that the finding meets the reporting criteria defined in the protocol.

Variants that do not meet this threshold will remain classified as VUS, will not be communicated as clinically positive findings, and may undergo follow-up or further analysis exclusively within the research context.

Changes made in the manuscript:

First, the final paragraph of the subsection “Untargeted metabolomics study” has been replaced with the following text:

“This complementary approach will enable the identification of biochemical abnormalities associated with variants of uncertain or incompletely characterized clinical significance and will provide additional evidence for their interpretation. In cases where a VUS is identified together with an untargeted metabolomic profile compatible with the disorder under evaluation, the metabolomic finding will be considered an additional source of evidence within the structured ACMG/AMP classification framework but will not, in isolation, determine variant reclassification or clinical reporting. Its applicability as phenotypic or functional evidence—for example, PP4 or PS3, respectively—and the strength assigned will be determined according to the disease mechanism, the specificity of the metabolic profile, and the degree of analytical and clinical validation of the assay, in accordance with ACMG/AMP and ClinGen recommendations. Each case will be reviewed by a multidisciplinary committee that will integrate the metabolomic findings with population, computational, segregation, phenotypic, functional, and case-level evidence. A variant will be reclassified as likely pathogenic and clinically communicated only when the combined independent evidence meets one of the ACMG/AMP combinations established for that category, no relevant contradictory evidence is present, and the finding meets the reporting criteria defined in the protocol. Variants that do not meet this threshold will remain classified as VUS, will not be communicated as clinically positive findings, and may undergo follow-up or further analysis exclusively within the research context.”

This modification appears on page 30, lines 725-747.

Second, the corresponding sentence in the Abstract, Methods section has been revised to avoid suggesting that metabolomics independently determines reclassification:

“Untargeted metabolomics using DBS will be applied in selected cases, particularly for prioritised variants of uncertain significance (VUS), to provide complementary biochemical evidence that may contribute to variant classification.”

This modification appears on page 3, lines 57-61.

**Major Comment 2**

**The reporting policy as described is insufficient for a clinical genetics protocol. The manuscript does not address several critical questions that will inevitably arise in clinical practice. It is unclear whether ACMG Secondary Findings (SF v3.2) will be disclosed and, if so, how adult-onset actionable findings will be handled when identified in newborns. The management of carrier status is not described specifically, whether single heterozygous pathogenic variants in autosomal recessive disease genes will be returned to families, a scenario that will be encountered frequently given the size of the gene panel. The manuscript does not specify how pharmacogenetic findings will be communicated or by whom, nor does it address whether P/LP variants in genes with incomplete penetrance will be reported and, if so, what clinical caveats will accompany disclosure.**

**Response:**
We thank the Reviewer for highlighting the need for a more explicit and comprehensive reporting policy. We agree that the previous version did not sufficiently distinguish between secondary findings, carrier status, pharmacogenetic results, and findings associated with incomplete penetrance.

ACMG secondary findings are not actively sought or reported because variant filtering, interpretation, and reporting are restricted to the predefined NBGenS disease and pharmacogenetic panels. Any subsequent analysis beyond the original virtual panel prompted by a relevant phenotype would constitute a separate clinically indicated investigation and would be managed according to the applicable clinical, ethical, consent, and ACMG secondary-findings procedures.

Isolated heterozygous pathogenic or likely pathogenic variants in genes associated with autosomal recessive diseases are not returned as carrier findings because they do not establish a diagnosis or require an immediate clinical intervention in the newborn.

Pathogenic or likely pathogenic variants associated with conditions showing incomplete penetrance are reported only when the gene–disease association is sufficiently established, the estimated penetrance is clinically relevant, and the finding enables preventive, surveillance, follow-up, or therapeutic measures that may benefit the child. Variant-, genotype-, gene-, and disease-level evidence, including age-, sex-, and modifier-dependent effects, is considered through individual multidisciplinary review.

Clinically actionable pharmacogenetic findings are communicated through a separate report prepared and validated by a healthcare professional with training in pharmacogenetics. The report is incorporated into the electronic health record of the Galician Health Service. Findings whose clinical relevance, analytical reliability, or actionability is insufficiently established are not returned.

The subsection “Genetic variants report” defines Potentially Reportable Findings (PRF) as pathogenic or likely pathogenic variants associated with a sufficiently established paediatric-onset disease, an available early intervention, and a zygosity consistent with the inheritance pattern. All other scenarios were classified as No Reportable Findings (NRF). Although this definition already excluded carrier findings and variants that did not meet the required penetrance or clinical-relevance thresholds, we acknowledge that these exclusions were not sufficiently explicit.

Changes made in the manuscript:

To clarify the NRF category, we have expanded its definition in the subsection “Genetic variants report” on page 22, beginning at line 541. The revised text reads:

“All other scenarios are classified as NRF, including the absence of reportable pathogenic or likely pathogenic variants; VUS, even when selected for untargeted metabolomics or subsequent reevaluation; isolated heterozygous pathogenic or likely pathogenic variants in genes associated with autosomal recessive diseases; and pathogenic or likely pathogenic variants associated with low, incomplete, variable, or insufficiently established penetrance, unless they are considered clinically relevant and enable preventive, surveillance, follow-up, or therapeutic measures with potential benefit for the child. These findings will not be communicated as clinically positive results, although they may be retained for subsequent research evaluation.

ACMG secondary findings are neither actively sought nor reported. Variant filtering, interpretation, and reporting are restricted to the predefined NBGenS disease and pharmacogenetic panels; variants outside these analytical scopes are excluded from review and therefore cannot be returned as secondary findings within the NBGenS workflow. Any subsequent analysis beyond the original panel prompted by the development of a relevant phenotype will be considered a separate clinically indicated investigation and managed according to the applicable clinical, ethical, consent, and ACMG secondary-findings procedures. For further details on the variant interpretation and reporting decision framework, please refer to S5 Fig.”

We have also clarified the communication of clinically actionable pharmacogenetic findings in the subsection “Secondary outcomes: Pharmacogenetic study” on page 26, lines 658-661, as follows:

“A healthcare professional with training in pharmacogenetics will communicate these findings to families through a separate report, which will be incorporated into the electronic health record of the Galician Health Service. Findings with insufficient clinical relevance, analytical reliability, or actionability will not be returned.”

**Major Comment 3**

**The reanalysis and recontact framework is also absent. The authors do not define what constitutes the trigger for variant reanalysis whether time-based, event-based, or reclassification-driven nor how genomic data will be managed across the 10-year retention period as gene-disease knowledge evolves. It is not stated whether families will be notified prior to data deletion at the end of the retention period. Finally, no framework is provided for handling variants in genes with variable expressivity, which is particularly relevant given that the panel encompasses 603 gene-disease pairs with heterogeneous penetrance profiles. A formal reporting decision algorithm addressing these scenarios is essential for a protocol paper and should be provided as a supplementary figure.**

**Response:**
We thank the Reviewer for this important comment. We agree that the protocol should define the distinction between variant reevaluation and case reanalysis, the triggers for reanalysis, recontact criteria, genomic data management, and the handling of findings associated with variable expressivity.

Variant reevaluation refers to reassessment of a previously identified variant in light of new evidence, whereas case reanalysis involves renewed interrogation of a participant’s genomic data using updated analytical methods, panel content, phenotypic information, and knowledge resources. A scheduled reanalysis will be performed 12 months after the initial analysis, followed by annual review of the panel and interpretation resources. Reanalysis may also be triggered by new clinical or phenotypic information, relevant methodological updates, new gene–disease or variant–disease evidence, updated clinical recommendations, or variant reclassification. Families will be recontacted only when the revised result meets the protocol reporting criteria and may have clinical implications. In all cases, the results will be documented.

We have also clarified that pseudonymized genomic data will be retained for ten years, initially in secure active storage and subsequently in secure archival storage. At the end of the retention period, data will be deleted. Families will be informed of this procedure during consent, and validated reports incorporated into the clinical record will remain subject to the applicable healthcare-documentation regulations.

For findings associated with variable expressivity, gene–disease pairs were classified into three groups: (i) those meeting all inclusion criteria; (ii) those meeting the criteria subject to specific interpretative or clinical conditions; and (iii) those associated with conditions also detectable through conventional biochemical newborn screening. The detailed allocation of individual gene–disease pairs to these groups is not shown in S3 Fig because it will remain subject to ongoing curation during subsequent panel updates. This classification will support panel documentation and review but will not, by itself, determine whether a finding is reportable. Reporting decisions will be based primarily on the evidence concerning the specific variant and will require a clinically relevant risk and an available beneficial clinical action.

These decision pathways have also been summarized in a new supplementary figure, entitled “S5 Fig. Decision algorithm for variant interpretation, reporting, reanalysis, and recontact in the NBGenS protocol.”. This figure has been added to the Supporting Information and is cited in the manuscript on line 487 and 558.

Changes made in the manuscript:

A clarifying paragraph has been added to the subsection “Variant analysis and interpretation” on pages 19-20, lines 475-482:

“A scheduled case reanalysis will be performed 12 months after the initial analysis. Thereafter, the virtual panel, gene–disease associations, interpretation resources, and relevant clinical recommendations will be reviewed annually throughout the data-retention period. Reanalysis may also be initiated at any time following new clinical, biochemical, metabolomic, or phenotypic information; a material update to the panel or bioinformatic workflow that may affect diagnostic sensitivity; new robust gene–disease or variant–disease evidence; new clinical recommendations; or reclassification of a previously identified variant with potential implications for diagnosis, prevention, surveillance, or treatment.

Families will be recontacted only when reanalysis identifies a new or modified result that meets the protocol reporting criteria and has potential clinical relevance. Reanalyses without clinically relevant changes will not be communicated individually, although their completion and outcome will be documented. For further details on the reanalysis triggers and family recontact criteria, please refer to S5 Fig.”

The information concerning genomic data management in the “Data collection” section has also been expanded on page 14, lines 331-340:

“Genomic data and derived analytical files will be stored in pseudonymized form with restricted access for ten years from their generation. During the active phase of the project, they will remain in secure active storage and, after project completion, will be transferred to secure archival storage while remaining available for protocol-authorized reanalysis. At the end of the retention period, data will be securely deleted. This procedure will not affect validated clinical reports incorporated into the electronic health record, which will be retained in accordance with applicable regulations. Families will be informed of the retention period and planned data disposition during the consent process; no additional notification before deletion or anonymization is planned.”

The “Gene panel” subsection has been expanded on page 17, lines 415-435, to clarify the classification of gene–disease pairs and, specifically, to include conditions associated with incomplete penetrance or variable expressivity within the second group, for which additional interpretative or clinical considerations are required:

“Starting from an initial list compiled from the literature, existing panels, and other relevant sources, gene–disease pairs were classified into three groups: (i) pairs that adequately met all predefined inclusion criteria; (ii) pairs that met these criteria but required consideration of specific interpretative or clinical conditions, such as incomplete penetrance or variable expressivity; and (iii) pairs associated with conditions also detectable through conventional biochemical newborn screening. The detailed allocation of gene–disease pairs to these three groups is not shown in S3 Table, as this classification will remain subject to ongoing curation during subsequent panel updates. This classification will support documentation and periodic review of the panel but will not, by itself, determine whether a finding is reportable. A core panel was established from these groups and will be periodically updated as new evidence emerges, allowing gene–disease pairs to be added, removed, or reclassified. This dynamic-panel approach will ensure flexibility and scalability while preserving the scientific consistency and predefined inclusion standards of the panel (S3 Table). The current version comprises 549 genes and 603 gene–disease pairs.”

To further clarify the management of these cases, we added an explanatory paragraph to the “Genetic variant reports” subsection on page 22 lines 539-541:

“For conditions showing variable expressivity, reporting decisions are based primarily on the evidence available for the specific variant and, when this is insufficient, on the corresponding genotype and gene–disease association.”

**Major Comment 4**

**The manuscript states that CNVs are detected using the DRAGEN pipeline but provides insufficient methodological detail for clinical evaluation. The minimum reportable CNV size, CNV classification criteria, and validation methodology are not described. It is not stated whether CNV analysis is restricted to genes within the virtual SNV panel or performed genome-wide the latter raises additional secondary findings considerations that would require separate ethical and clinical governance. How intragenic CNVs are classified and reported is also unaddressed. CNV detection from WES has well-documented sensitivity limitations compared to array-based approaches, and the analytical validation of the exome-based CNV calling pipeline must be explicitly described.**

**Response:**
We thank the Reviewer for identifying the insufficient methodological description of the CNV workflow. We agree that the original manuscript did not provide enough information to evaluate its analytical scope, interpretation criteria, validation strategy, or clinical reporting policy. The Methods section will therefore include the following clarification.

Candidate CNVs will be identified using two complementary read-depth approaches: the DRAGEN germline CNV pipeline (v4.2.7), with copy-number calling enabled and normalization against a panel of normal samples, and GATK-gCNV workflow based on a cohort model. Read-count intervals for GATK-gCNV will be derived from the exome-capture targets using PreprocessIntervals with a bin length of 0 and 250-bp padding. The minimum unit evaluated will therefore be one captured target, generally corresponding to one exon.

Clinical interpretation and reporting will be restricted to CNVs affecting genes included in the virtual panel. Off-panel calls will be retained only for quality-control or research purposes and will not be returned to families. Consequently, CNV analysis will not create a separate genome-wide secondary-findings pathway.

CNVs affecting panel genes will be classified according to the ACMG/ClinGen technical standards for constitutional CNVs, supported by ClassifyCNV, AnnotSV, and ClinGen dosage-sensitivity curation. Final classification will remain subject to expert review. Intragenic single- and multi-exon CNVs will be evaluated according to the dosage sensitivity of the affected gene and their predicted effect on the reading frame.

CNVs detected concordantly by both callers will be prioritized as high-confidence candidates. Every potentially reportable CNV will require confirmation using an appropriate orthogonal method, such as multiplex ligation-dependent probe amplification for intragenic events or chromosomal microarray analysis for larger or more complex events, before clinical communication. The manuscript will also state explicitly that WES-based CNV calling has lower and less uniform sensitivity than dedicated copy-number methods, particularly for small, single-exon, intronic, and complex events.

Changes made in the manuscript:

The following clarification will be included in the Methods section on pages 15-16, lines 376-394:

“CNV candidates will be generated using the provider’s DRAGEN germline CNV pipeline (v4.2.7)(42), with copy-number calling enabled and panel-of-normals normalization, together with an in-house GATK-gCNV workflow based on a cohort model(43). For GATK-gCNV, read-count intervals will correspond to the exome-capture targets and will be generated using PreprocessIntervals with a bin length of 0 and 250-bp padding. GATK-gCNV calls will be retained at a quality score ≥100 and DRAGEN calls at FILTER PASS. Two calls will be considered concordant, and therefore a high-confidence candidate, when they show ≥50% reciprocal overlap and affect the same gene in the same dosage direction (loss or gain); calls supported by a single caller will be reviewed but not classified as high-confidence. Calls larger than 3 Mb will be discarded as capture-driven artefacts, leaving an effective resolution down to a single captured target or exon.

The analytical performance of the combined workflow will be evaluated before clinical reporting to establish the validated minimum reportable range, particularly for single-exon events. CNV calling will be performed across the captured exome regions, but clinical interpretation and reporting will be restricted to genes included in the virtual panel. Off-panel CNVs will not be returned to families. Analytical performance will be estimated against reference materials with known copy-number states and previously array- or MLPA-confirmed in-house cases, reporting sensitivity and positive predictive value stratified by event size to define the minimum reportable range.

Panel CNVs will be classified according to the 2019 ACMG/ClinGen constitutional CNV framework(44), supported by ClassifyCNV(45), AnnotSV, and ClinGen dosage-sensitivity curations. Intragenic single- and multi-exon CNVs will be assessed according to gene dosage sensitivity and their predicted effect on the reading frame.

Concordant calls from both approaches will be considered high-confidence candidates. Clinically actionable CNVs will be confirmed using Multiplex Ligation-dependent Probe Amplification (MLPA) or chromosomal microarray analysis before communication to the family. The limitations of WES-based CNV detection, particularly for small, single-exon, intronic, or poorly captured events, will be considered when interpreting negative results.”

**Major Comment 5**

**The pharmacogenetic secondary analysis requires additional methodological detail. The list of pharmacogenes included and the CPIC evidence level required for inclusion are not specified. The methodology for star-allele calling from WES data is not described, despite the fact that WES-based pharmacogenomics has recognized limitations for complex loci such as CYP2D6, where CNV and pseudogene interference are well-established challenges, and CYP2C19, where structural variants may be missed. Whether orthogonal confirmation is planned for clinically actionable calls must be stated. Finally, the clinical context and professional responsible for communicating pharmacogenetic results to families must be defined, as this requires expertise distinct from that of the neonatology or clinical genetics team.**

**Response:**
We thank the Reviewer for this important comment. We have expanded the pharmacogenetic Methods and Table S7 to cover the points raised.

Star alleles will be inferred from aligned WES CRAM files using PyPGx v0.26 across the 88 pharmacogenes supported by the software. This workflow will combine statistical haplotype phasing with copy-number estimation based on normalised read depth for CNV-defined loci, including CYP2D6 and UGT1A1. The remaining genes will be diplotyped from the joint-genotyped VCF using PharmCAT v3.2.0, while HLA-B57:01 and HLA-A31:01 will be typed using HLA-LA. Candidate gene–drug pairs will be selected from PharmVar, the CDC Tier 1 genomic applications, the AEMPS pharmacogenomic biomarker database, and PharmGKB. The resulting research universe will comprise 1,160 gene–drug associations involving 81 genes and 464 drugs, as detailed in S7 Table.

Reporting will be organised into two tiers. The clinical tier will be restricted to CPIC Level A actionable pairs involving 22 genes for which prescribing guidance is available from CPIC, DPWG, or the FDA. Within this tier, priority will be given to associations with PharmGKB evidence levels 1A–2A and to AEMPS-authorised medicines with paediatric relevance. Lower-evidence, non-paediatric, or non-AEMPS-covered associations will be included in an exploratory tier for possible future reanalysis but will not be returned under the current protocol.

The protocol will explicitly acknowledge the limitations of exon-capture WES for complex pharmacogenetic loci. In particular, WES may not reliably resolve CYP2D6–CYP2D7 hybrid alleles, whole-gene deletions or duplications, or pseudogene interference, and rare CYP2C19 structural variants may be missed. If PyPGx or a paralogue-aware caller such as Cyrius does not provide a reliable CYP2D6 copy-number or structural call, no clinically actionable CYP2D6 structural result will be issued from WES alone. All clinically actionable pharmacogenetic findings will be confirmed using an appropriate orthogonal method, such as a TaqMan copy-number or genotyping assay, long-range PCR, Sanger sequencing, or a validated pharmacogenetic genotyping panel, before communication.

As clarified in the response to Major Comment 2, clinically actionable pharmacogenetic findings will be communicated to families by a healthcare professional with training in pharmacogenetics through a separate report, which will be incorporated into the electronic health record of the Galician Health Service. Findings with insufficient clinical relevance, analytical reliability, or actionability will not be returned.

Changes made in the manuscript:

The following clarification will be included in the Methods section on pages 26-27, lines 636-665:

“Star alleles will be inferred from aligned WES CRAM files using PyPGx v0.26 for the pharmacogenes supported by the software. Statistical haplotype phasing will be combined with copy-number estimation based on normalised read depth for CNV-defined loci, including CYP2D6 and UGT1A1. Genes not resolved using PyPGx will be diplotyped from the joint-genotyped VCF using PharmCAT v3.2.0, while HLA-B57:01 and HLA-A31:01 will be typed using HLA-LA.

Pharmacogenetic findings will be organised into clinical and exploratory tiers. The clinical tier will be restricted to CPIC Level A actionable gene–drug pairs involving 22 genes for which prescribing guidance from CPIC, DPWG, or the FDA is available. Within this tier, priority will be given to associations with PharmGKB evidence levels 1A–2A and to AEMPS-authorised medicines with paediatric relevance. Associations with lower levels of evidence, without paediatric relevance, or involving medicines not covered by the AEMPS criteria will be retained for possible future reanalysis but will not be returned under this protocol.

The recognised limitations of exon-capture WES at complex pharmacogenetic loci will be considered during interpretation. In particular, WES may not reliably resolve CYP2D6–CYP2D7 hybrid alleles, whole-gene deletions or duplications, or pseudogene interference, and rare CYP2C19 structural variants may be missed. If a reliable CYP2D6 copy-number or structural call cannot be obtained, no clinically actionable structural result will be issued from WES data alone.

All clinically actionable pharmacogenetic findings will undergo orthogonal confirmation using an appropriate targeted method, such as a TaqMan copy-number or genotyping assay, long-range PCR, Sanger sequencing, or a validated pharmacogenetic genotyping panel, before communication.

***

This secondary analysis will highlight the considerable versatility and potential utility of the genomic information that will be collected at the individual level, enabling both alternative analytical approaches and future reanalysis that may prove clinically valuable should new indications arise during the study period.”

**Major Comment 6**

**The manuscript briefly acknowledges WES limitations in the Discussion; however, a clinical genetics protocol should address these explicitly in the Methods, as they directly affect diagnostic yield and the scope of reportable findings. Repeat expansion disorders including Friedreich ataxia, fragile X syndrome, and myotonic dystrophy are not reliably detected by standard WES pipelines but may be represented in the gene panel. Mitochondrial genome coverage from WES is variable and insufficient for reliable clinical mitochondrial disease diagnosis. Deep intronic variants affecting splicing, an increasingly recognized class of pathogenic variation, are largely missed by exon-capture approaches. Low-level somatic or mosaic variants may also fall below standard variant-calling thresholds. These limitations should be explicitly acknowledged in the Methods section and their implications for diagnostic yield estimates addressed.**

**Response:**
We thank the Reviewer for this important observation. We agree, and we now address these limitations explicitly in the Methods, with a note on how each one affects diagnostic yield.

Standard WES will not be considered reliable for the definitive detection of pathogenic repeat expansions. As a targeted complementary analysis, panel genes containing known pathogenic repeat regions will be screened using ExpansionHunter v5 and a curated variant catalogue applied to the WES reads. These findings will be considered screening-level results only. If clinical findings or family history suggest a repeat expansion disorder, such as Friedreich ataxia, fragile X syndrome, or myotonic dystrophy, the participant will be referred for an appropriate targeted assay, such as repeat-primed PCR or Southern blotting, irrespective of the WES findings.

Because mitochondrial genome coverage from exon-capture WES will be variable, mitochondrial DNA variants will only be assessed at positions meeting predefined depth and quality requirements. When mitochondrial disease is clinically suspected, dedicated mitochondrial DNA testing will be considered rather than relying on WES alone.

Variants located near captured splice sites will be evaluated using SpliceAI and Pangolin with predefined thresholds. However, most deep intronic pathogenic variants will fall outside the captured regions and will therefore not be reliably detected. When clinical suspicion remains high despite a negative WES result, genome sequencing or RNA-based testing may be considered.

Low-level somatic or mosaic variants may fall below standard variant-calling thresholds. Mosaic-sensitive tools, including DeepMosaic and MosaicForecast, will be used to support the identification of low variant-allele-fraction candidates. Nevertheless, analysis of blood alone will not exclude tissue-restricted mosaicism.

These variant classes will consequently remain underdetected by WES, and their contribution to the diagnostic yield will be interpreted as a lower-bound estimate. Cases with strong clinical suspicion and negative or inconclusive WES findings will be considered for appropriate targeted or orthogonal testing.

Changes made in the manuscript:

The following clarification will be included in the Methods section on pages 20-21, lines 488–520:

“Standard WES will not be considered reliable for the definitive detection of pathogenic repeat expansions. As a targeted complementary analysis, panel genes containing known pathogenic repeat regions will be screened using ExpansionHunter v5 and a curated variant catalogue applied to the WES reads. These calls will be treated as screening-level findings only. If clinical findings or family history suggest a repeat expansion disorder, such as Friedreich ataxia, fragile X syndrome, or myotonic dystrophy, an appropriate targeted assay, including repeat-primed PCR or Southern blotting, will be considered irrespective of the WES findings.

Mitochondrial DNA variants will only be assessed at positions meeting predefined depth and quality requirements because mitochondrial genome coverage from exon-capture WES will be variable. If mitochondrial disease is clinically suspected, dedicated mitochondrial DNA sequencing will be considered rather than relying on WES alone. Mitochondrial variants will be reported only at positions with a minimum read depth of 100× and base and mapping quality ≥20, and heteroplasmy will be quantified and reported at variant-allele fractions ≥10%.

Variants located near captured splice sites will be evaluated using SpliceAI and Pangolin with predefined thresholds. However, most deep intronic pathogenic variants will not be detected because they fall outside the captured regions. Genome sequencing or RNA-based testing may therefore be considered when clinical suspicion remains high despite a negative WES result. The predefined thresholds will be a SpliceAI or Pangolin delta score ≥0.20 as supporting evidence of a splicing effect, ≤0.10 as evidence against, and 0.10–0.20 as uninformative; for canonical splice-site positions without an exact delta score, a dbscSNV ada or rf score >0.6 will be used as a fallback.

Mosaic-sensitive tools, including DeepMosaic and MosaicForecast, will be used to support the identification of low variant-allele-fraction candidates. Nevertheless, low-level mosaic variants may remain below the detection threshold, and analysis of blood will not exclude tissue-restricted mosaicism. This analysis will target variant-allele fractions of approximately 1–30%, will be restricted to genes with neonatal-actionable mosaic phenotypes, and will require manual inspection of every candidate.

Repeat expansions, mitochondrial variants, deep intronic variants, and low-level mosaic variants will consequently remain underdetected by WES. Diagnostic yield estimates for these variant classes will therefore be interpreted as lower-bound estimates, and cases with strong clinical suspicion and negative or inconclusive WES findings may undergo appropriate targeted or orthogonal testing.”

**Minor Comments**

**Minor Comment 1** **— Untargeted metabolomics**

**The UM component is a methodological strength of this study. However, the statistical thresholds defining a reportable metabolomic abnormality are not specified the authors should state the p-value, fold-change, or z-score cutoff used relative to controls. The approach for distinguishing disease-specific metabolic signals from nonspecific perturbations common in neonates including those related to prematurity, intercurrent illness, or dietary variation must be described.**

**Response:**
We thank the Reviewer for this important comment and have expanded the methodological description in the manuscript. Metabolic abnormalities are defined by comparing metabolite levels in each case with those observed in a reference control cohort using Z-score calculations. Metabolites with a Z-score ≥ +2 or ≤ −2 are considered potentially abnormal. To ensure reproducibility and consistency, an automated bioinformatic pipeline has been developed to perform data processing, Z-score calculation, and the initial identification of altered metabolites.

Regarding the distinction between disease-specific metabolic signals and nonspecific alterations commonly observed during the neonatal period, interpretation is not based solely on statistical thresholds. All metabolomic profiles are reviewed by a multidisciplinary team with expertise in clinical metabolomics and inherited metabolic disorders, which assesses the biological relevance of the findings in the clinical context of each participant. Potential confounding factors are considered during this review, including prematurity, gestational age, nutritional status, concomitant medical treatments, and intercurrent illness, as well as metabolic patterns previously described in these settings. Only abnormalities that are consistent with the pathophysiological mechanism of the disorder under evaluation and cannot be reasonably explained by these confounding factors are considered supportive evidence for clinical interpretation.

The corresponding changes have been incorporated into the Methods section through the addition of a clarifying paragraph on pages 28-29, lines 695–714:

“Data analysis will be performed using SCIEX OS software in combination with an in-house–developed automated bioinformatic pipeline. Metabolite levels in each participant will be compared with those observed in a reference control cohort using Z-scores, and values ≥ +2 or ≤ −2 will be considered potentially abnormal. The pipeline will perform data processing, Z-score calculation, and the initial identification of altered metabolites. All metabolomic profiles will subsequently be reviewed by a multidisciplinary team with expertise in clinical metabolomics and inherited metabolic disorders. This review will consider potential confounding factors, including prematurity, gestational age, nutritional status, concomitant medical treatments, intercurrent illness, and metabolic patterns previously described in these settings. Only abnormalities consistent with the pathophysiological mechanism of the disorder under evaluation and not reasonably explained by these potential confounders will be considered supportive evidence for clinical interpretation. The reference control cohort will comprise unaffected newborns analysed on the same platform and matched for gestational and postnatal age, feeding, and analytical batch. Because a |Z| ≥ 2 threshold will flag a proportion of features by chance across the untargeted profile, an isolated Z-score will not be treated as evidence in itself: only alterations that are biologically consistent with the candidate variant and reproducible will be retained, and no metabolite will contribute as ACMG evidence on the basis of the Z-score alone.”

**Minor Comment 2-9 — Additional minor issues**

**Response to tense standardization:**

We thank the Reviewer for this observation. We have reviewed the entire manuscript and corrected inconsistencies in verb tense throughout the text. Present and future tenses are now used consistently for protocol procedures, whereas the past tense is restricted to activities that had already been completed, such as ethics approval and the initial gene-panel curation.

**Response to Reference 42:**

We thank the Reviewer for identifying this citation error. The incorrect reference has been replaced with the appropriate citation to the current ACGS Best Practice Guidelines for Variant Classification in Rare Disease 2024. The corresponding entry has also been corrected in the reference list: <https://www.acgs.uk.com/media/12533/_media_12533_uk-practice-guidelines-for-variant-classification-v12-2024.pdf>

**Response to reference numbering and incomplete references:**

We thank the Reviewer for identifying these bibliographic inconsistencies. We corrected the numbering error that resulted in reference number 52 appearing twice, when the second occurrence should have been numbered 62. We also added the missing bibliographic information to reference 62.

In addition, the entire reference list was reviewed, and other incomplete entries, duplicated references, and numbering inconsistencies were corrected and standardized.

**Response to “VUS hot variants”:**

We thank the Reviewer for this observation and agree that the term “VUS HOT” does not correspond to standard ACMG/ClinGen terminology. In this study, the term was used operationally to identify variants of uncertain significance prioritized for evaluation by untargeted metabolomics. Because analytical capacity and available resources do not allow functional evaluation of every VUS, prioritization criteria were required.

Accordingly, “VUS prioritized for untargeted metabolomics” are defined as variants that, following initial classification according to ACMG/AMP and ClinGen recommendations, could potentially reach the likely pathogenic category if untargeted metabolomics provided additional applicable phenotypic or functional evidence, such as PP4 or PS3. This strategy therefore focuses on variants with a higher pretest probability of clinical relevance and for which metabolomic findings could materially contribute to the final classification.

To avoid confusion with standard nomenclature, we have replaced the term “VUS HOT” with “VUS prioritized for untargeted metabolomics” in Table 3 (page 27, line 680) and added the corresponding definition to the Methods section (pages 27, lines 672–677).

“Because available resources and analytical capacity do not permit untargeted metabolomic evaluation of every VUS, variants will be prioritized for this analysis. VUS prioritized for untargeted metabolomics are defined as variants that, following initial classification according to ACMG/AMP and ClinGen recommendations, could potentially reach the likely pathogenic category if metabolomic findings provided additional applicable phenotypic or functional evidence, such as PP4 or PS3.”

**Response to the expression “prior to start the enrolment”:**

We thank the Reviewer for identifying this grammatical error. The expression has been replaced with “prior to commencing enrolment” on line 896.

**Response to wording and turnaround time:**

We thank the Reviewer for this suggestion. The wording has been revised on lines 216–218 to replace the approximate single-value estimate with a more precise description of the projected turnaround-time distribution, reporting the expected median and 90th percentile.

“The projected median time from recruitment to report delivery is 1 month, with a 90th percentile of 2 months.”

**Response to the BabyDetect comparison:**

We thank the Reviewer for this valuable suggestion. We agree that BabyDetect represents a particularly relevant methodological comparator for NBGenS. We have therefore expanded the Discussion to compare the design, analytical strategy, implementation framework, and methodological trade-offs of both studies. The added comparison appears on lines 861–893:

“In this regard, the integration of UM with genomic and conventional biochemical screening will provide an important complementary advantage (68,69), offering biochemical evidence that may help refine variant interpretation and reduce uncertainty. ScreenPlus (70) illustrates the value of multitiered strategies combining biochemical assays with targeted molecular test-ing. However, BabyDetect (15) represents a closer methodological comparator for NBGenS, as both are prospective, single-centre studies embedded in a public maternity setting and evaluate genomic screening as a first-tier approach for severe, actionable paediatric disor-ders. BabyDetect demonstrated high population acceptance and identified clinically relevant conditions that had not been detected through conventional NBS, thereby providing empirical support for the added diagnostic reach of genomic screening.

The BabyDetect experience also illustrates several methodological trade-offs that are directly relevant to NBGenS. Its targeted sequencing strategy relied on a predefined capture panel and on automated filtering of previously curated pathogenic or likely pathogenic variants, followed by expert review. Although this approach substantially reduced the number of variants requiring manual assessment, a previously unreported pathogenic TJP2 variant was not initially flagged, and modification of the targeted panel required a new analytical validation process. In comparison, NBGenS will use WES with a dynamically curated virtual panel, allowing the analysed gene set to be updated without redesigning the sequencing assay, while retaining expert multidisciplinary review of potentially reportable findings. NBGenS will also extend the implementation framework by using residual DBS collected through routine NBS and by incorporating selected UM analyses, pharmacogenetics, psychosocial assessment, clinical follow-up, and health-economic evaluation. These differences will allow the study to examine whether a broader and more adaptable workflow can be integrated into an established public NBS programme while maintaining appropriate interpretative and reporting safeguards.”

**Response to AI-supported reclassification and gene-panel updating:**

We thank the Reviewer for this clarification. We agree that the original wording could overstate the role of automated and artificial-intelligence-based systems. We have therefore moderated these statements to make clear that such systems are intended as decision-support tools rather than substitutes for expert evaluation. Final variant-classification, gene-curation, inclusion, exclusion, and reporting decisions remain subject to expert multidisciplinary review. These revisions appear on lines 855–860 and 903–906:

“Currently, comprehensive reanalysis requires substantial manual effort and time, highlighting the potential value of automated and AI-assisted tools to support evidence retrieval, variant prioritization, and standardization of the reanalysis process, while final classification and re-porting decisions will remain subject to expert multidisciplinary review.”

“Future development of AI-supported systems for continuous evidence aggregation and panel updating, as demonstrated in other similar initiatives (73,74), may represent a sustainable decision support for expert curation, while final inclusion, exclusion, and reporting decisions will remain subject to multidisciplinary review.”

**Additional clarification regarding Reviewer #1, Question 4**

Thank you for highlighting this point. We have revised the Data Availability statement to clarify that this protocol does not report study outcome data and that the data supporting the future findings will be made available with the publication of the study results or through an appropriate repository.

The following statement has been included on pages 2, lines 32-35:

“This protocol article does not report or analyse study outcome data. Upon completion of the study, the data supporting the findings will be made available with the results publication or through an appropriate repository.”

**Closing statement**

We once again thank the Editor and the Reviewers for their careful evaluation and constructive feedback. We believe that the revisions made in response to their comments have substantially improved the clarity, methodological rigour, and overall quality of the manuscript. We trust that the revised version adequately addresses all the issues raised and hope that it will now be considered suitable for publication in PLOS ONE.

Sincerely,

MARIA LUZ COUCE PICO
On behalf of all authors
