# Supplementary material for: The NBGENS project protocol: Pilot study for genetic newborn screening in Galicia
Source: PLoS One. 2026 Oct 8;21(10):e0359270. doi: 10.1371/journal.pone.0359270 (PMC13649029; doi:10.1371/journal.pone.0359270)
Supplement: Peer review history

# Peer review: Decision Letter 0

**DOI:** 10.1371/journal.pone.0359270.r001

**Authors:** Muhammad Farooq

14 Jul 2026

Dear Dr. Couce,

Thank you for submitting your manuscript to PLOS ONE. After careful consideration, we feel that it has merit but does not fully meet PLOS ONE’s publication criteria as it currently stands. Therefore, we invite you to submit a revised version of the manuscript that addresses the points raised during the review process.

Please submit your revised manuscript by Aug 28 2026 11:59PM. If you will need more time than this to complete your revisions, please reply to this message or contact the journal office at plosone@plos.org. When you're ready to submit your revision, log on to https://www.editorialmanager.com/pone/ and select the 'Submissions Needing Revision' folder to locate your manuscript file.

- A letter that responds to each point raised by the academic editor and reviewer(s). You should upload this letter as a separate file labeled 'Response to Reviewers'.
- A marked-up copy of your manuscript that highlights changes made to the original version. You should upload this as a separate file labeled 'Revised Manuscript with Track Changes'.
- An unmarked version of your revised paper without tracked changes. You should upload this as a separate file labeled 'Manuscript'.

If you would like to make changes to your financial disclosure, please include your updated statement in your cover letter. Guidelines for resubmitting your figure files are available below the reviewer comments at the end of this letter.

If applicable, we recommend that you deposit your laboratory protocols in protocols.io to enhance the reproducibility of your results. Protocols.io assigns your protocol its own identifier (DOI) so that it can be cited independently in the future. For instructions see: https://journals.plos.org/plosone/s/submission-guidelines#loc-laboratory-protocols. Additionally, PLOS ONE offers an option for publishing peer-reviewed Lab Protocol articles, which describe protocols hosted on protocols.io. Read more information on sharing protocols at https://plos.org/protocols?utm_medium=editorial-email&utm_source=authorletters&utm_campaign=protocols.

As the corresponding author, your ORCID iD is verified in the submission system and will appear in the published article. PLOS supports the use of ORCID, and we encourage all coauthors to register for an ORCID iD and use it as well. Please encourage your coauthors to verify their ORCID iD within the submission system before final acceptance, as unverified ORCID iDs will not appear in the published article. *Only*  the individual author can complete the verification step; PLOS staff *cannot*  verify ORCID iDs on behalf of authors.

We look forward to receiving your revised manuscript.

Kind regards,

Muhammad Farooq

Academic Editor

PLOS One

Journal Requirements:

When submitting your revision, we need you to address these additional requirements.

1. Please ensure that your manuscript meets PLOS ONE's style requirements, including those for file naming. The PLOS ONE style templates can be found at https://journals.plos.org/plosone/s/file?id=wjVg/PLOSOne_formatting_sample_main_body.pdf and https://journals.plos.org/plosone/s/file?id=ba62/PLOSOne_formatting_sample_title_authors_affiliations.pdf

2. We note that the grant information you provided in the ‘Funding Information’ and ‘Financial Disclosure’ sections do not match.

When you resubmit, please ensure that you provide the correct grant numbers for the awards you received for your study in the ‘Funding Information’ section.

3. Thank you for stating the following financial disclosure:

[Carlos III Health Institute (ISCIII by PI23/00276). CIBERER (Convocatoria Proyectos de Investigacion Traslacional 2023)].

Please state what role the funders took in the study.  If the funders had no role, please state: "The funders had no role in study design, data collection and analysis, decision to publish, or preparation of the manuscript."

If this statement is not correct you must amend it as needed.

Please include this amended Role of Funder statement in your cover letter; we will change the online submission form on your behalf.

4. Please remove any funding-related text from the manuscript. Please note that funding information should not appear in any section or other areas of your manuscript. We will publish the funding statement that is present in the Financial Disclosure section of the online submission form.

5. Thank you for providing your underlying data as Supporting Information.

We note that the data set contains text or data that is not in English. Please note that PLOS is an English-language publisher, so we require data sets to be provided in English as well. Please upload an English-language version of your data set.

This will also allow us to determine if your data follows PLOS standards per our Data Availability policy here: https://journals.plos.org/plosone/s/data-availability

6. We note that Figure S2 in your submission contains copyrighted images. All PLOS content is published under the Creative Commons Attribution License (CC BY 4.0), which means that the manuscript, images, and Supporting Information files will be freely available online, and any third party is permitted to access, download, copy, distribute, and use these materials in any way, even commercially, with proper attribution. For more information, see our copyright guidelines: http://journals.plos.org/plosone/s/licenses-and-copyright.

We require you to either (1) present written permission from the copyright holder to publish these figures specifically under the CC BY 4.0 license, or (2) remove the figures from your submission:

1. You may seek permission from the original copyright holder of Figure S2 to publish the content specifically under the CC BY 4.0 license.

We recommend that you contact the original copyright holder with the Content Permission Form (http://journals.plos.org/plosone/s/file?id=7c09/content-permission-form.pdf) and the following text:

“I request permission for the open-access journal PLOS ONE to publish XXX under the Creative Commons Attribution License (CCAL) CC BY 4.0 (http://creativecommons.org/licenses/by/4.0/). Please be aware that this license allows unrestricted use and distribution, even commercially, by third parties. Please reply and provide explicit written permission to publish XXX under a CC BY license and complete the attached form.”

Please upload the completed Content Permission Form or other proof of granted permissions as an "Other" file with your submission.

In the figure caption of the copyrighted figure, please include the following text: “Reprinted from [ref] under a CC BY license, with permission from [name of publisher], original copyright [original copyright year].”

2. If you are unable to obtain permission from the original copyright holder to publish these figures under the CC BY 4.0 license or if the copyright holder’s requirements are incompatible with the CC BY 4.0 license, please either i) remove the figure or ii) supply a replacement figure that complies with the CC BY 4.0 license. Please check copyright information on all replacement figures and update the figure caption with source information. If applicable, please specify in the figure caption text when a figure is similar but not identical to the original image and is therefore for illustrative purposes only.

If the reviewer comments include a recommendation to cite specific previously published works, please review and evaluate these publications to determine whether they are relevant and should be cited. There is no requirement to cite these works unless the editor has indicated otherwise.

[Note: HTML markup is below. Please do not edit.]

Reviewers' comments:

Reviewer's Responses to Questions

**Comments to the Author**

1. Does the manuscript provide a valid rationale for the proposed study, with clearly identified and justified research questions?

Reviewer #1: Partly

**********

2. Is the protocol technically sound and planned in a manner that will lead to a meaningful outcome and allow testing the stated hypotheses?

Reviewer #1: Partly

**********

3. Is the methodology feasible and described in sufficient detail to allow the work to be replicable?

Reviewer #1: Yes

**********

4. Have the authors described where all data underlying the findings will be made available when the study is complete?

The PLOS Data policy

Reviewer #1: No

**********

5. Is the manuscript presented in an intelligible fashion and written in standard English?

Reviewer #1: Yes

**********

Please use the space provided to explain your answers to the questions above and, if applicable, provide comments about issues authors must address before this protocol can be accepted for publication. You may also include additional comments for the author, including concerns about research or publication ethics.

You may also provide optional suggestions and comments to authors that they might find helpful in planning their study.

Reviewer #1: The NBGenS protocol describes a clinically important and timely pilot study integrating whole-exome sequencing (WES), untargeted metabolomics (UM), pharmacogenetics, psychosocial assessment, and health economic analysis into a public newborn screening (NBS) program in Spain. The multidisciplinary design and integration of UM within an established public health NBS framework are genuine strengths that distinguish this study from prior genomic NBS initiatives. However, from a clinical genetics standpoint, several fundamental issues in variant interpretation, reporting framework, gene panel design, and ethical governance require substantive revision before this protocol can be considered methodologically sound.

Major Comments

1. The authors state: "In cases where abnormalities associated with a VUS are detected, the variant would be reclassified as likely pathogenic and reported." This statement is clinically incorrect and inconsistent with ACMG/AMP variant classification guidelines (Richards et al., Genet Med 2015), which the authors simultaneously cite as their interpretive framework.

Under ACMG/AMP criteria, variant reclassification from VUS to likely pathogenic requires integrated assessment of multiple evidence streams: population frequency data, in silico predictions, segregation analysis, case-level evidence, and functional data. A single metabolomic signal constitutes functional evidence under the PS3 criterion but is insufficient in isolation to support reclassification. Applying this policy as written risks significant over-reporting with direct clinical consequences for asymptomatic newborns and their families, and could cause clinical harm through false-positive reporting in a screening context.

The authors must revise this section to explicitly state that UM findings represent one line of evidence within a structured ACMG/AMP classification framework, describe the multidisciplinary variant review process that will govern reclassification decisions, and specify which combination of ACMG criteria must be met before a VUS is upgraded to likely pathogenic.

2. The reporting policy as described is insufficient for a clinical genetics protocol. The manuscript does not address several critical questions that will inevitably arise in clinical practice. It is unclear whether ACMG Secondary Findings (SF v3.2) will be disclosed and, if so, how adult-onset actionable findings will be handled when identified in newborns. The management of carrier status is not described specifically, whether single heterozygous pathogenic variants in autosomal recessive disease genes will be returned to families, a scenario that will be encountered frequently given the size of the gene panel. The manuscript does not specify how pharmacogenetic findings will be communicated or by whom, nor does it address whether P/LP variants in genes with incomplete penetrance will be reported and, if so, what clinical caveats will accompany disclosure.

3.The reanalysis and recontact framework is also absent. The authors do not define what constitutes the trigger for variant reanalysis whether time-based, event-based, or reclassification-driven nor how genomic data will be managed across the 10-year retention period as gene-disease knowledge evolves. It is not stated whether families will be notified prior to data deletion at the end of the retention period. Finally, no framework is provided for handling variants in genes with variable expressivity, which is particularly relevant given that the panel encompasses 603 gene-disease pairs with heterogeneous penetrance profiles. A formal reporting decision algorithm addressing these scenarios is essential for a protocol paper and should be provided as a supplementary figure.

4. The manuscript states that CNVs are detected using the DRAGEN pipeline but provides insufficient methodological detail for clinical evaluation. The minimum reportable CNV size, CNV classification criteria, and validation methodology are not described. It is not stated whether CNV analysis is restricted to genes within the virtual SNV panel or performed genome-wide the latter raises additional secondary findings considerations that would require separate ethical and clinical governance. How intragenic CNVs are classified and reported is also unaddressed. CNV detection from WES has well-documented sensitivity limitations compared to array-based approaches, and the analytical validation of the exome-based CNV calling pipeline must be explicitly described.

5. The pharmacogenetic secondary analysis requires additional methodological detail. The list of pharmacogenes included and the CPIC evidence level required for inclusion are not specified. The methodology for star-allele calling from WES data is not described, despite the fact that WES-based pharmacogenomics has recognized limitations for complex loci such as CYP2D6, where CNV and pseudogene interference are well-established challenges, and CYP2C19, where structural variants may be missed. Whether orthogonal confirmation is planned for clinically actionable calls must be stated. Finally, the clinical context and professional responsible for communicating pharmacogenetic results to families must be defined, as this requires expertise distinct from that of the neonatology or clinical genetics team.

6. The manuscript briefly acknowledges WES limitations in the Discussion; however, a clinical genetics protocol should address these explicitly in the Methods, as they directly affect diagnostic yield and the scope of reportable findings. Repeat expansion disorders including Friedreich ataxia, fragile X syndrome, and myotonic dystrophy are not reliably detected by standard WES pipelines but may be represented in the gene panel. Mitochondrial genome coverage from WES is variable and insufficient for reliable clinical mitochondrial disease diagnosis. Deep intronic variants affecting splicing, an increasingly recognized class of pathogenic variation, are largely missed by exon-capture approaches. Low-level somatic or mosaic variants may also fall below standard variant-calling thresholds. These limitations should be explicitly acknowledged in the Methods section and their implications for diagnostic yield estimates addressed.

Minor Comments

The UM component is a methodological strength of this study. However, the statistical thresholds defining a reportable metabolomic abnormality are not specified the authors should state the p-value, fold-change, or z-score cutoff used relative to controls. The approach for distinguishing disease-specific metabolic signals from nonspecific perturbations common in neonates including those related to prematurity, intercurrent illness, or dietary variation must be described.

Several additional minor issues require attention. First, tense is used inconsistently throughout the manuscript. Multiple sections shift between past tense, present tense and future tense within and across sections, including the Abstract, Methods, and Discussion. This is a protocol paper describing a prospective study; present or future tense is appropriate for procedures not yet completed, while past tense should be reserved for completed steps such as ethics approval and panel curation. The authors should standardize tense throughout the manuscript.

Reference 42 is cited in the context of the ACGS Best Practice Guidelines for Variant Classification but appears to link to Karczewski et al. (gnomAD, Nature 2020) these are distinct documents, and this appears to be a citation error requiring correction. The reference list also contains numbering inconsistencies: reference 54 appears twice, and reference 62 is missing author and title information.

The term "VUS hot variants" is used as a trigger for UM eligibility in Table 3 but is not defined anywhere in the manuscript and does not correspond to standard ACMG/ClinGen nomenclature. This term must be defined at first use or replaced with accepted standard terminology.

The phrase "prior to start the enrolment" should read "prior to commencing enrolment." Turnaround time is described throughout as "approximately one month"; for a protocol paper this should be expressed as a projected median or mean with anticipated range.

The Discussion benchmarks NBGenS against ScreenPlus but omits comparison with BabyDetect (Boemer et al., Nat Med 2025, reference 15), which is the most methodologically analogous study and is already cited in the Introduction. This comparison should be included in the Discussion. Finally, the Discussion implies that AI systems may enable automated variant reclassification; this claim should be tempered, as current AI tools support but do not replace expert clinical review, and overstating their role is inappropriate in a protocol paper intended to inform national health policy.

**********

what does this mean?). If published, this will include your full peer review and any attached files.

If you choose “no”, your identity will remain anonymous but your review may still be made public.

**Do you want your identity to be public for this peer review?**  For information about this choice, including consent withdrawal, please see our Privacy Policy

Reviewer #1: No

**********

[NOTE: If reviewer comments were submitted as an attachment file, they will be attached to this email and accessible via the submission site. Please log into your account, locate the manuscript record, and check for the action link "View Attachments". If this link does not appear, there are no attachment files.]

To ensure your figures meet our technical requirements, please review our figure guidelines: https://journals.plos.org/plosone/s/figures

You may also use PLOS’s free figure tool, NAAS, to help you prepare publication quality figures: https://journals.plos.org/plosone/s/figures#loc-tools-for-figure-preparation.

NAAS will assess whether your figures meet our technical requirements by comparing each figure against our figure specifications.

# Author comment: Author response to Decision Letter 1

**DOI:** 10.1371/journal.pone.0359270.r002

11 Aug 2026

The responses to the editorial and reviewers requests have been included in the "Response to Reviewers" document uploaded with this revised submission.

Attachment Submitted filename: Rebuttal_letter_NBGENS.docx

# Peer review: Decision Letter 1

**DOI:** 10.1371/journal.pone.0359270.r003

**Authors:** Muhammad Farooq

13 Sep 2026

The NBGENS project protocol: pilot study for genetic Newborn screening in Galicia

PONE-D-26-17272R1

Dear Dr. Maria Luz Couce,

We’re pleased to inform you that your manuscript has been judged scientifically suitable for publication and will be formally accepted for publication once it meets all outstanding technical requirements.

Within one week, you’ll receive an e-mail detailing the required amendments. When these have been addressed, you’ll receive a formal acceptance letter and your manuscript will be scheduled for publication.

An invoice will be generated when your article is formally accepted. Please note, if your institution has a publishing partnership with PLOS and your article meets the relevant criteria, all or part of your publication costs will be covered. Please make sure your user information is up-to-date by logging into Editorial Manager at Editorial Manager® and clicking the ‘Update My Information' link at the top of the page. For questions related to billing, please contact billing support.

If your institution or institutions have a press office, please notify them about your upcoming paper to help maximize its impact. If they’ll be preparing press materials, please inform our press team as soon as possible -- no later than 48 hours after receiving the formal acceptance. Your manuscript will remain under strict press embargo until 2 pm Eastern Time on the date of publication. For more information, please contact onepress@plos.org.

Kind regards,

Muhammad Farooq

Academic Editor

PLOS One

Additional Editor Comments (optional):

Reviewers' comments:

Reviewer's Responses to Questions

**Comments to the Author**

1. Does the manuscript provide a valid rationale for the proposed study, with clearly identified and justified research questions?

Reviewer #2: Yes

**********

2. Is the protocol technically sound and planned in a manner that will lead to a meaningful outcome and allow testing the stated hypotheses?

Reviewer #2: Yes

**********

3. Is the methodology feasible and described in sufficient detail to allow the work to be replicable?

Reviewer #2: Yes

**********

4. Have the authors described where all data underlying the findings will be made available when the study is complete?

The PLOS Data policy

Reviewer #2: No

**********

5. Is the manuscript presented in an intelligible fashion and written in standard English?

Reviewer #2: Yes

**********

Please use the space provided to explain your answers to the questions above and, if applicable, provide comments about issues authors must address before this protocol can be accepted for publication. You may also include additional comments for the author, including concerns about research or publication ethics.

You may also provide optional suggestions and comments to authors that they might find helpful in planning their study.

Reviewer #2: The authors have adressed the comments of reviewers 1 and revised the manuscript accordingly. The manuscript is well written.

**********

what does this mean?). If published, this will include your full peer review and any attached files.

If you choose “no”, your identity will remain anonymous but your review may still be made public.

**Do you want your identity to be public for this peer review?**  For information about this choice, including consent withdrawal, please see our Privacy Policy

Reviewer #2: No

**********

# Editor report: Acceptance letter

**DOI:** 10.1371/journal.pone.0359270.r004

**Authors:** Muhammad Farooq

PONE-D-26-17272R1

PLOS One

Dear Dr. Couce,

I'm pleased to inform you that your manuscript has been deemed suitable for publication in PLOS One. Congratulations! Your manuscript is now being handed over to our production team.

At this stage, our production department will prepare your paper for publication. This includes ensuring the following:

* All references, tables, and figures are properly cited

* All relevant supporting information is included in the manuscript submission,

* There are no issues that prevent the paper from being properly typeset

You will receive further instructions from the production team, including instructions on how to review your proof when it is ready. Please keep in mind that we are working through a large volume of accepted articles, so please give us a few days to review your paper and let you know the next and final steps.

Lastly, if your institution or institutions have a press office, please let them know about your upcoming paper now to help maximize its impact. If they'll be preparing press materials, please inform our press team within the next 48 hours. Your manuscript will remain under strict press embargo until 2 pm Eastern Time on the date of publication. For more information, please contact onepress@plos.org.

You will receive an invoice from PLOS for your publication fee after your manuscript has reached the completed accept phase. If you receive an email requesting payment before acceptance or for any other service, this may be a phishing scheme. Learn how to identify phishing emails and protect your accounts at https://explore.plos.org/phishing.

If we can help with anything else, please email us at customercare@plos.org.

Thank you for submitting your work to PLOS One and supporting open access.

Kind regards,

PLOS One Editorial Office Staff

on behalf of

Dr. Muhammad Farooq

Academic Editor

PLOS One
